# Antimicrobial Effectiveness of *Ribes nigrum* L. Leaf Extracts Prepared in Natural Deep Eutectic Solvents (NaDESs)

**DOI:** 10.3390/antibiotics13121118

**Published:** 2024-11-22

**Authors:** Maria-Beatrice Solcan, Ana-Maria Vlase, Gabriel Marc, Dana Muntean, Tibor Casian, George Cosmin Nadăș, Cristiana Ștefania Novac, Daniela-Saveta Popa, Laurian Vlase

**Affiliations:** 1 Department of Toxicology, Faculty of Pharmacy, Iuliu Hatieganu University of Medicine and Pharmacy, 8 Victor Babeș Street, 400012 Cluj-Napoca, Romania; solcan.maria.beatrice@elearn.umfcluj.ro (M.-B.S.); dpopa@umfcluj.ro (D.-S.P.); 2 Department of Pharmaceutical Botany, Faculty of Pharmacy, Iuliu Hatieganu University of Medicine and Pharmacy, 8 Victor Babeș Street, 400012 Cluj-Napoca, Romania; 3 Department of Pharmaceutical Chemistry, Faculty of Pharmacy, Iuliu Hatieganu University of Medicine and Pharmacy, 8 Victor Babeș Street, 400012 Cluj-Napoca, Romania; marc.gabriel@umfcluj.ro; 4 Department of Pharmaceutical Technology and Biopharmacy, Faculty of Pharmacy, Iuliu Hatieganu University of Medicine and Pharmacy, 8 Victor Babeș, Street, 400012 Cluj-Napoca, Romania; dana.muntean@umfcluj.ro (D.M.); casian.tibor@umfcluj.ro (T.C.); laurian.vlase@umfcluj.ro (L.V.); 5 Department of Microbiology, Faculty of Veterinary Medicine, University of Agricultural Sciences and Veterinary Medicine, 3-5 Calea Mănăștur, 400372 Cluj-Napoca, Romania; gnadas@usamvcluj.ro (G.C.N.); cristiana.novac@usamvcluj.ro (C.Ș.N.)

**Keywords:** *Ribes nigrum* L., polyphenols, procyanidins, hydrogen-bond acceptor, hydrogen-bond donor, antimicrobial activity, antioxidant potential, chlorogenic acid, ultrasound-assisted extraction, ultra-turrax extraction

## Abstract

Background: Blackcurrant (*Ribes nigrum* L.) leaves are valuable sources of bioactive compounds, including phenolic acids, flavonoids, and tannins, which contribute to their potent antioxidant, anti-inflammatory, and antimicrobial properties. Objectives: The overall aim of this study was to investigate the antimicrobial potential of extracts rich in bioactive compounds from blackcurrant leaves prepared in natural deep eutectic solvents (NaDESs). The objectives included the optimization of polyphenols extraction in NaDESs, characterization of the phytochemical composition by liquid chromatography–mass spectrometry (LC-MS), explanation of the chemical interactions between solvent systems and the main bioactive compound (chlorogenic acid) by molecular dynamics simulations, and evaluation of biological efficacy through antimicrobial tests. Methods: Two hydrogen-bond acceptors (HBAs) and three hydrogen-bond donors (HBDs) were tested. The experimental design included variables such as the HBA:HBD molar ratio, water percentage, extraction time, and extraction techniques used, specifically ultrasound-assisted extraction (UAE) and ultra-turrax extraction (UTE). The evaluated responses included the total polyphenol content, total flavonoid content, and total antioxidant activity. Antimicrobial assays were performed on four Gram-positive and three Gram-negative bacterial species, as well as one fungus, *Candida albicans*. Results: The extracts obtained by UAE showed higher concentrations of polyphenols and increased antioxidant potential. LC-MS analysis revealed the predominant presence of chlorogenic acid. The extracts showed significant activities against Gram-positive bacteria and *Candida albicans*. Conclusions: This study highlights the antioxidant and antimicrobial potentials of blackcurrant leaves extracts prepared in NaDESs, confirming that this type of solvent enhances polyphenols extraction and offers perspectives for new therapeutic formulations.

## 1. Introduction

Berries are appreciated worldwide for their nutritional values and high contents of vitamins, minerals, and compounds with antioxidant actions [1]. Among these, blackcurrant (*Ribes nigrum* L.) is particularly valued not only for its nutritious berries but also for its leaves, which are valuable sources of bioactive compounds. Recent studies have shown that blackcurrant leaves, because of their rich polyphenol content, are more effective than the berries [2] in reducing inflammation and protecting against oxidative stress. Additionally, they may offer protective benefits against microbial infections and inhibit the multiplication of certain viruses according to preclinical studies [3].

Polyphenols are hormetic compounds and secondary metabolites, their synthesis being stimulated by plants’ stressful conditions [4]. Because of their beneficial health effects, polyphenols are used as supplements and substitutes for synthetic additives in food production. The variation in these compounds is significantly influenced by the leaf position on the branch and the harvest date [4,5]. For maintaining the quality of the leaves, monitoring and controlling pests are important because studies have shown that infestations by certain pests can alter the polyphenolic composition of the leaves, thereby affecting their quality and therapeutic value [6]. Microbial contamination of plant raw materials, including leaf extracts of *Ribes nigrum* prepared in natural deep eutectic solvents (NaDESs), can significantly affect the quality of the final products intended for pharmaceutical and nutraceutical applications. Microorganisms, such as bacteria and fungi, can degrade essential bioactive compounds, such as polyphenols, thereby reducing the antioxidant activities and therapeutic efficacies of the extracts [7]. These contaminations not only compromise chemical stability but also pose a risk to the safety of end users, especially in pharmaceutical formulations [8]. Polyphenolic compounds are of interest because of their beneficial effects on health, being used both in dietary supplements and as substitutes for synthetic additives in food production, as well as bioactive molecules in the pharmaceutical and cosmetic industries. Similar to other plants, such as the oil palm (*Elaeis guineensis* Jacq.), where secondary metabolites play crucial roles in defense against diseases, in blackcurrant, these compounds are equally vital for maintaining the quality and therapeutic value of the leaves [9,10].

To guarantee accurate and reproducible results, the analysis of bioactive compounds from natural sources involves sample collection, pretreatment, extraction, and purification of the compounds, followed by their identification and quantification through various instrumental techniques [11]. The protocol for the selection and characterization of plant materials has been detailed by several researchers, including Kellogg et al. [12]. The pretreatment of samples includes grinding and homogenization and may involve air-drying or lyophilization [13]. The extraction method and type of solvent used play important roles in the yields and efficiencies for obtaining polyphenolic compounds. Traditional techniques, such as maceration and Soxhlet extraction, used for extraction from *Ribes nigrum*, frequently use organic solvents, like methanol [14], ethanol [15], and acetone [2], sometimes combined with water in various proportions [16,17]. But these organic compounds are known for their high volatility, flammability, and toxicity.

Deep eutectic solvents (DESs) were first synthesized in 2003 in the United Kingdom. They are accessible, inexpensive, and, most importantly, biodegradable and recyclable solvents. A DES consists of a hydrogen-bond acceptor (HBA) and a hydrogen-bond donor (HBD). Although the two substances that are mixed are crystalline at room temperature, their mixture is a liquid [18]. Unlike conventional solvents (e.g., ethanol, methanol, hexane, and butane), DESs are non-volatile, meaning they have very low vapor pressures and are therefore difficult to ignite [18]. Examples of DESs include choline chloride and urea, malic acid, and glucose. NaDESs have attracted particular attention because of their versatility in the extraction of bioactive compounds from plant materials. NaDESs represent a significant advancement in green chemistry, offering an efficient and environmentally friendly alternative to conventional solvents. They have been described as a combination of natural compounds, such as organic acids, sugars, or amino acids, which form a network of hydrogen bonds similar to those of classical DESs but with superior safety and biocompatibility profiles [18]. NaDESs have become a major subject of interest for researchers, as evidenced by the significant increase in the number of studies published on this topic. Over 60 reviews have documented the use of NaDESs in various fields, such as drug discovery [19], the creation of new materials [20], chromatographic analyses [21,22], and organic syntheses [23], among others [24]. Recent research suggests that NaDESs can improve the solubility and extraction efficiencies of bioactive compounds, as well as the stabilities of extracts over time [15]. Additionally, the use of NaDESs can contribute to reducing negative environmental impacts [25].

According to current knowledge, no studies have been reported in the literature regarding the use of NaDESs in obtaining extracts from *Ribes nigrum* leaves. Therefore, this study aims to explore the effectiveness of these solvents in the extraction of bioactive compounds, thus making an important contribution to the field of research. Although numerous studies have been conducted to optimize the extraction, isolation, and separation of polyphenols from natural sources, their highly diverse and complex structures make it impossible to adopt a universal method for the extraction of all plant polyphenols [26]. Conventional extraction methods have several significant disadvantages, such as a long process duration, high costs due to large solvent and energy consumption, low selectivity, risk of compound degradation due to prolonged heating, negative environmental impact, and issues related to inadequate solvent recycling [27]. In this context, many of these disadvantages can be mitigated or even eliminated by adopting more efficient techniques, such as ultrasound-assisted extraction (UAE) [2,15], microwave-assisted extraction (MAE) [16,28], or enzyme-assisted extraction (EAE) [29]. In recent years, these modern extraction methods have been developed to improve the yield and quality of bioactive compounds from plants, such as anthocyanins from blackcurrant [30].

Recent studies have investigated modern extraction methods for bioactive compounds from *Ribes nigrum*, such as UAE [30,31,32] and the use of the turbo-extraction (UTE) [32]. These methods provide eco-friendly alternatives to traditional maceration extraction. These methods have proven to be particularly effective. UAE utilizes ultrasound-induced cavitation to enhance mass transfer, allowing for the extraction of anthocyanins at low temperatures and shorter extraction times, minimizing the degradation of sensitive compounds [33]. Additionally, EAE employs specific enzymes to degrade cell walls, facilitating the release of anthocyanins without the need for large quantities of chemical solvents [29]. These methods not only improve yield but also help to preserve the sensory properties of the products, making them valuable for various industrial applications. MAE uses the interaction of microwaves with polar compounds, generating heat and disrupting cell walls, which allows for more efficient solvent penetration and rapid extraction of bioactive compounds from plants [34]. UTE using the ultra-turrax homogenizer has been successfully applied in recent years for the efficient isolation of bioactive compounds from various plant matrices. It ensures a high dispersion of plant material in the solvent due to its high rotational speed and strong shear forces, achieving similar quality tinctures in much shorter time compared to traditional methods like maceration. The extraction process is highly efficient, often taking just a few minutes, but extended time may negatively impact thermolabile compounds due to increased temperature [32].

The objectives of the present study were as follows: (1) to optimize the extraction conditions of bioactive compounds from *Ribes nigrum* using NaDESs and two modern extraction methods, UAE and UTE, to maximize the polyphenol content and antioxidant activity; (2) to evaluate the antimicrobial activity of NaDES-derived extracts, with a view toward the development of new topical formulations for human use.

## 2. Results

### 2.1. Design of Experiments

#### 2.1.1. Outcomes from the Experimental Matrix and Fitting the Data with the Models

The experimental design matrix, consisting of 39 experiments carried out on blackcurrant leaves, was developed using Modde software, version 13.0.2. The results from these experiments are detailed below. Table 1 displays the factors hypothesized to influence the extraction yield, including the types of HBA and HBD, their molar ratio, water content in the NaDESs, extraction method, and duration. Additionally, it contains the investigated responses, such as the total phenolic content (TPC), total flavonoid content (TFC), and total antioxidant activity (TAA) measured by the 2,2-diphenyl-1-(2,4,6-trinitrophenyl) hydrazine (DPPH) assay. This table provides an overview of the results from the experimental runs, highlighting that these factors had varying degrees of impact—both positive and negative—on the observed outcomes.

The performance of the fitted models for TPC, TFC, and TAA along with the results of the ANOVA test on the significance of the corresponding models are provided in Table 2 and Table 3.

As illustrated in Table 2, the high R^2^ values (>0.85) indicate that the model explains a substantial proportion of the variation in the response. Additionally, the Q^2^ value over 0.55 confirms that the model’s predictive ability is adequate. The model’s validity is further supported by values above 0.4, while the reproducibility of the results is very good, with values greater than 0.7. This suggests that the variance in the response under identical experimental conditions is consistently well managed.

The *p*-values for ANOVA regression are below 0.05, indicating that the variation in the response is explained by factor variation. Furthermore, the *p*-values for the ANOVA lack of fit are greater than 0.05, suggesting that there is no lack of fit in the dataset.

#### 2.1.2. The Influence of Experimental Conditions on Dependent Variables

**Figure 1:**
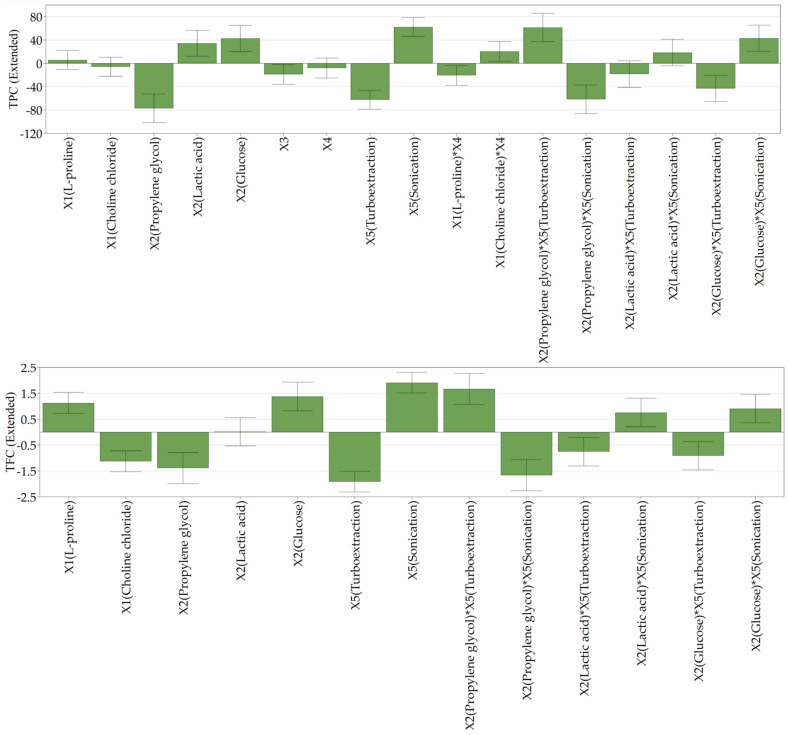

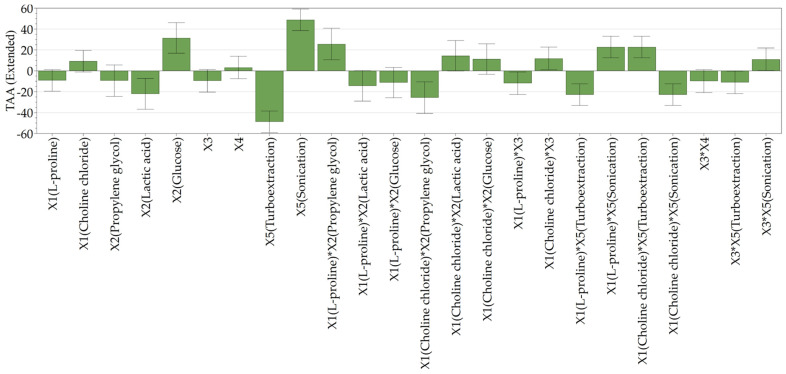
The influence of working conditions on the total phenolic content (TPC) and total flavonoid content (TFC) recovery and total antioxidant activity (TAA) from blackcurrant leaves, depicted as scaled and centered coefficient plots. X_1_—hydrogen-bond acceptor (HBA); X_2_—hydrogen-bond donor (HBD); X_3_—molar ratio HBA:HBD; X_4_—water ratio in deep eutectic solvent mixture; X_5_—extraction method; X_6_—extraction time.

Figure 1 illustrates the influence of the independent factors upon the responses (TPC, TFC, and TAA) as centered and scaled coefficients along with interactions between certain factors.

The use of glucose (X_2_) as the HBD as well as ultrasound-assisted extraction (X_5_) leads to higher TPC values. Similarly, a higher TFC is obtained by using L-proline (X_1_) as the HBA, glucose (X_2_) as the HBD, and ultrasound-assisted extraction (X_5_). A statistically significant interaction between X_2_ and X_5_ was observed for both evaluated responses. The interaction graph shows that the TPC and TFC values increase when lactic acid or glucose are used in combination with UAE. In contrast, the use of propylene glycol does not give the same results, the difference between the two extraction methods being smaller (minimal effect especially for TPC).

The statistical significance of the factors studied, including both their positive and negative effects upon the recovery of each identified and quantified phytochemical, has been assessed and is summarized in Table 4.

The results summarized in Table 4 suggest that the effect of X_1_ (HBA) presents an influence on the individual phytochemical extraction yield to a lesser extent compared to X_2_ (HBD), with an equal number of positive and negative effects observed (11 each). This variation highlights the importance of selecting the appropriate HBA type according to the specific phytochemical of interest. On the other hand, the effect of X_2_ (HBD) is significant, with both positive and negative effects, indicating that the choice of HBD should also be carefully made, considering the targeted phytochemical. The effect of X_3_ (combination ratio) was significant in only three cases, all with a positive influence, suggesting that increasing the combination ratio from one to two improves the extraction of three bioactive compounds determined by HPLC-MS, namely gentisic acid, hyperoside, and quercitrin. Similarly, the X_4_ effect (water ratio) was positive and significant in only six cases, indicating that a higher water content enhances the extraction of six compounds from blackcurrant leaves (gentisic acid, isoquercitrin, quercitrin, quercetol, kaempferol, procyanidin B2). X_5_ (extraction method) had a significant impact on most of the quantified phytochemicals, with some compounds being better extracted using UAE while others presented a better recovery with UTE. The effect of the last investigated factor, X_6_ (extraction time), was significant for five bioactive compounds, with increased extraction time being advantageous for four compounds (hyperoside, kaempferol, protocatechuic acid, and procyanidin B2) and disadvantageous for one (isoquercitrin). Details of the individual bioactive compounds whose extraction yield was statistically significantly influenced by the evaluated independent factors can be found in Supplementary Table S1. Therefore, quantitative factors have a minimal impact, making the optimization of the extraction process quite challenging. The probability of identifying optimal conditions for all investigated bioactive compounds from blackcurrant leaves is quite low, as it heavily depends on the specific phytochemicals targeted for optimization.

#### 2.1.3. Evaluation of Optimal Experimental Conditions to Obtain Extracts Rich in Phytochemicals

In the optimization stage, the main objective was to maximize the extraction of TPC and to achieve significant antioxidant activity (evaluated as TAA). To obtain the optimal extract, according to the Modde software, the extraction conditions should include the use of L-proline as the HBA and lactic acid as the HBD, in a ratio of 1124:1, with approximately 50% water content, and the use of sonication (UAE) for 5 min. The results for the optimal extracts together with the recovery rates are presented in Table 5.

### 2.2. Phenolic Content, Flavonoid Content, and Antioxidant Activity

Table 1 shows the results obtained in the experimental design applied in this study. It can be observed how the independent variables—TPC, TFC, and TAA-DPPH—varied depending on the composition of the deep eutectic solvent mixture, the percentage of water in the solvent, the extraction method, and the extraction time.

In the case of UTE, the TPC of blackcurrant leaf extracts ranged from 74.37 ± 0.04 (sample N7) to 353.20 ± 0.09 (sample N11) mg GAE/g dw. UAE was significantly more efficient in extracting polyphenolic compounds, and TPC ranged from 153.03 ± 0.05 (sample N22) to 490.53 ± 0.10 (sample N37) mg GAE/g dw. The high content of polyphenolic compounds in the leaves of blackcurrant, an autochthonous variety from the wild mountain flora of the northern region of Romania, is remarkable.

The ability of NaDESs to extract flavonoids from blackcurrant leaves also differs, depending on their composition and extraction method. Thus, in extracts prepared by UTE, the TFC ranged from 2.95 ± 0.00 (sample N7) to 9.07 ± 0.04 (sample N4) mg QE/g dw, while in the case of extracts obtained by UAE it was between 4.93 ± 0.02 (sample N22) and 14.55 ± 0.04 (sample N32) mg QE/g dw.

The antioxidant activity of the extracts, as reflected by the DPPH radical scavenging capacity, was significantly higher in UAE than in UTE. Only 4 extracts out of 19 prepared by UTE had values >200 mg TE/g dw, whereas only 3 extracts out of 19 obtained by UAE had values <200 mg TE/g dw. Surprisingly, the lowest antioxidant activity, 8.86 ± 2.01 mg TE/g dw, recorded for sample N8 (NaDES: L-Pro:LA, 2:1 molar ratio, 50% water, UTE, 10 min), does not correlate with the content of polyphenolic compounds, this extract having relatively average values of TPC (218.37 ± 0.06 mg GAE/g dw) and TFC (6.92 ± 0.01 mg QE/g dw). This suggests that the free radical scavenging activity may be inhibited or modulated by interactions occurring between or with deep eutectic solvent components. The highest antioxidant activity was recorded for sample N36: 263.13 ± 0.01 mg TE/g dw (NaDES: choline chloride:glucose (ChChl:Glu), 1:1 molar ratio, 50% water, UAE, 10 min).

To identify the optimal conditions for extracting bioactive compounds from blackcurrant leaves, a factorial experimental design was conducted. This design varied several parameters, including the extraction method, extraction time, HBA molar ratio, and water percentage, to determine the impact of each variable on extraction efficiency. Table 1 presents the detailed results of these experiments.

Several remarkable results were obtained in this study. The highest total phenolic content was achieved in experiment N37, with an impressive value of 490.53 mg GAE/g dw, indicating the high efficiency of the conditions used in this experiment for phenolic extraction. Regarding the total flavonoid content, experiment N32 recorded the highest value, at 14.55 mg QE/g dw, suggesting that the specific parameters of this experiment are the most suitable for flavonoid extraction. Additionally, the highest total antioxidant activity was observed in experiment N36, with a value of 263.13 mg TE/g dw, demonstrating the maximum efficacy of these conditions in enhancing the antioxidant activity of the extracts.

These results highlight the importance of optimizing extraction conditions to obtain maximum quantities of valuable bioactive compounds from blackcurrant leaves. Table 1 provides a detailed overview of the experimental variables, and the results obtained, facilitating the identification of the most efficient extraction conditions.

### 2.3. Phytochemical Analysis by LC–MS

Table 6, Table 7 and Table 8 present the quantification results for individual phenolic acids, flavanols, and flavonoids identified in the analyzed extracts from blackcurrant leaves.

In this study, several remarkable results were obtained through LC-MS analysis. The highest content of gentisic acid was found in sample N31, with a value of 11.45 µg/g dw, while the highest content of caffeic acid was observed in sample N33, with 129.78 µg/g dw. Sample N39 exhibited the highest chlorogenic acid content, with 3942.01 µg/g dw, and sample N20 had the highest content of 4-*O*-caffeoylquinic acid, with 21.14 µg/g dw, and *p*-coumaric acid, with 12.14 µg/g dw. The highest gallic acid content was found in sample N1, with 12.39 µg/g dw, protocatechuic acid in sample N33, with 28.21 µg/g dw, and vanillic acid in sample N2, with 0.94 µg/g dw.

In terms of flavanols, sample N24 presented the highest contents of epicatechin (36.58 µg/g dw), procyanidin B2 (B2) (15.02 µg/g dw), procyanidin C2 (C2) (19.97 µg/g dw), and procyanidin C1 (C1) (217.76 µg/g dw). Sample N26 was remarkable for having the highest contents of catechin (11.92 µg/g dw) and procyanidin A1 (A1) (34.30 µg/g dw). The highest content of epigallocatechin (EGC) was found in sample N19, with 43.03 µg/g dw, while the highest epigallocatechin gallate (EGCG) content was observed in sample N6, with 36.31 µg/g dw. The highest procyanidin B3 (B3) content was recorded in sample N34, with 42.48 µg/g dw, procyanidin B3 (B3) in sample N38, with 25.17 µg/g dw, and procyanidin B4 (B4) in sample N25, with 19.52 µg/g dw.

Regarding flavonols, the highest hyperoside content was found in sample N24, with 216.59 µg/g dw, while the highest isoquercetin content was observed in sample N15, with 466.39 µg/g dw. Sample N19 had the highest rutin content, with 126.01 µg/g dw, while sample N20 had the highest contents of quercitrin (230.85 µg/g dw), quercetol (12.43 µg/g dw), and kaempferol (7.05 µg/g dw). The highest kaempferol-3-*O*-rhamnoside content was recorded in sample N2, with 9.13 µg/g dw.

LC-MS analysis of blackcurrant leaf extracts revealed the presence of three classes of polyphenolic compounds: phenolic acids (Table 6), flavanols (Table 7), and flavonols (Table 8).

Two hydroxycinnamic acids were identified and quantified from the phenolic acid category: *p*-coumaric acid (concentrations between 0 and 12.14 µg/g dw) and caffeic acid (between 0 and 129.78 µg/g dw), and two isomers of an ester of caffeic acid, 3-*O*-caffeoylquinic acid, often called chlorogenic acid (between 21.64 and 3992.21 µg/g dw), and 4-*O*-caffeoylquinic acid, also called cryptochlorogenic acid (between 0 and 21.14 µg/g dw), respectively. Ferulic acid, sinapic acid, and caftaric acid were not detected in any of the extracts analyzed. Chlorogenic acid, which could be quantified in all the extracts prepared and analyzed, was the major phenolic acid in all extracts, being extractable mainly by UAE (Table 6).

Among the hydroxybenzoic acids, gallic acid (between 0.14 and 12.39 µg/g dw) and protocatechuic acid (between 0.07 and 28.21 µg/g dw) were presented in all extracts in quantifiable amounts, while syringic acid was not detected in any of the analyzed extracts. Vanillic acid (between 0 and 0.94 µg/g dw) was present in only some of the extracts, and gentisic acid (2,5-dihydroxybenzoic acid), an isomer of protocatechuic acid (3,4-dihydroxybenzoic acid), although identified in all extracts, could only be quantified in 6 samples (between 0 and 11.45 µg/g dw) out of the 39 extracts.

Flavonoids are a class of polyphenols with remarkable antioxidant activity. They share the C6-C3-C6 diphenyl-propane backbone structure, in which the two C6 rings (A and B rings) present phenolic groups. These are usually linked to a heterocyclic ring (C ring), which is often a closed pyran. The structural diversity of flavonoids is determined by the type of hydroxylation, the state of oxidation, and the variation in the chromane C ring. Thus, flavonoids are classified in many subgroups as flavonols, flavanols, proanthocyanidins, anthocyanidins, flavones, chalcones, and isoflavones.

The LC-MS methods used in flavonoid analysis revealed the presence of four flavanols, seven proanthocyanidins (Table 7), and seven flavonols (Table 8) in blackcurrant leaf extracts.

All extracts contained epicatechin (between 0.07 and 36.58 µg/g dw) and EGC (between 1.41 and 43.03 µg/g dw). Compounds such as catechin (between 0.07 and 11.92 µg/g dw) and EGCG (between 0 and 0.63 µg/g dw) (Table 7) were identified only in certain extracts and in lower amounts. From the category of procyanidins, four isomers of procyanidin B (B1–B4) were identified and quantified in all extracts, the most abundant being procyanidin B2 (between 0.06 and 150.05 µg/g dw), followed by procyanidin C1 (between 0 and 217.76 µg/g dw). Procyanidins A1 (maximum 34.30 µg/g dw) and C2 (maximum 19.97 µg/g dw) were present in lower quantities and only in some extracts prepared by UAE (Table 7). Again, the much higher extraction yield for procyanidins from blackcurrant leaves in the presence of NaDESs is registered for UAE compared to UTE.

From the flavonol class, the aglycones quercetin and kaempferol, the most commonly found in the plant kingdom [35], were present in variable amounts. Quercetin was detected in certain extracts and quantified only in four samples, in concentrations ranging from 4.45 to 12.43 µg/g dw. Kaempferol was detected in all samples, but in most of them the amount was below the limit of quantification of the analytical method (<LOQ) (maximum concentration determined: 7.05 µg/g dw) (Table 8). Among the heterosides, kaempferol-3-*O*-rhamnoside was found only in trace amounts (<LOQ) or not detected (ND). In contrast, the extracts were abundant in quercetin heterosides: isoquercetin (ranging from 60.31 to 466.39 µg/g dw), hyperoside (ranging from 4.09 to 216.59 µg/g dw), quercitrin (ranging from 28.00 to 230.85 µg/g dw, with three extracts obtained via UAE showing no detection for this compound), and rutin (not detected in one UAE extract, <LOQ in most UAE extracts, and ranging from 42.14 to 126.01 µg/g dw in the others). Notably, higher levels of isoquercetin and rutin were observed in the extracts prepared by UTE, while hyperoside levels were elevated in those obtained through UAE.

Therefore, the extraction methods applied in this experimental design show different extraction efficiencies depending on the phytochemical compound. The blackcurrant leaves used in the study are particularly rich in chlorogenic acid and quercetin heterosides such as isoquercetin, hyperoside, quercitrin, and rutin.

### 2.4. Results of Molecular Dynamics Simulations

Molecular dynamics simulations were carried out to assess the influence of the mixture components’ ratio on the intermolecular interactions of chlorogenic acid as a solute model, with the solvation system. As the HBA, two types of molecules were used—L-proline and choline chloride—while as the HBD three types of molecules were used—propylene glycol, *β*-D-glucose, and an equimolecular ratio of R-lactic acid:L-lactic acid. To respect the ratio of the components from the extraction mixtures in the simulation box, the number of molecules of each component was calculated to respect the respective ratio.

Regarding the chemical features of the HBA molecules which were used, a significant difference between them was identified. While L-proline is an amino acid, possessing an acidic carboxyl group and a pyrrolidine ring containing a secondary amine, choline chloride is a quaternary ammonium salt with a primary alcohol group. From this point of view alone, the molecular interaction of chlorogenic acid with HBA molecules can be expected to be much more complex than simple hydrogen bonding.

On the other hand, the HBD molecules which were used have at least one alcohol group. While propylene glycol and *β*-D-glucose are both polyols, lactic acid has a carboxylic acid group.

Because of the complexity of interactions between the components of eutectic mixtures, a series of parameters were evaluated after the molecular dynamics. First, the hydrogen bonding was evaluated, because the chlorogenic acid taken as solute model may interact with the HBA, HBD, or water from the eutectic mixtures via this mechanism. Theoretically, strong hydrogen bonding between the solute and the eutectic solvent components can enhance solvation, leading to increased solubility of the solute. On the other hand, if the solute molecules do not have suitable hydrogen-bonding groups, if the interactions between the solute and the eutectic solvent components are weak, or the interaction between the components of the solute are too strong, the solubility of the compounds may be reduced. Given the complexity of the phenomenon, an analysis was carried out on the radial distribution function and the quantification of the interaction energy between chlorogenic acid and the solvent mixtures to obtain a clearer image of the interactions.

In the analysis of the hydrogen bonding between chlorogenic acid and the components of the systems comprised of L-proline, propylene glycol, and water (systems 1, 2, 3, 4, 20, and 21), a standout was the slightly smaller total number of hydrogen bonds (6.29 bonds) in system 1, which was comprised by the highest ratio of L-proline. In the case of systems 1, 2, 3, 4, 20, and 21, no obvious connection can be made between the composition of the system and the number of hydrogen bonds. In the systems comprised by the mixture of L-proline, *β*-D-glucose, and water (systems 14, 15, 16, 32, and 33), a very good connection between the ratio of components and the hydrogen-bonding pattern to the respective components can be identified. It seems that the interaction is mainly dependent on the ratio of *β*-D-glucose (R^2^ = 0.90) and water (R^2^ = 0.92), less so than on the proline ratio (R^2^ = 0.59). The highest number of average total hydrogen bonds was found for systems 15 (8.85 bonds) and 33 (7.31 bonds). For the systems comprised of L-proline, lactic acid, and water mixtures (systems 7–9, 25, and 25–26), the hydrogen bonding to the respective components seems to be independent of the ratio of L-proline (R^2^ = 0.04), but it seems to be driven by the ratio of lactic acid (R^2^ = 0.66) and water (R^2^ = 0.65). Detailed data regarding the analysis of the hydrogen bonding between chlorogenic acid and the systems using L-proline as the HBA are presented in Table 9.

Concerning the hydrogen-bonding analysis between chlorogenic acid and systems using choline chloride as the HBA, the details are summarized in Table 10. In the systems comprised of mixtures between choline chloride, propylene glycol, and water (systems 5, 6, 22, 23, and 24), the highest correlation between the ratio of components and the hydrogen bonding was identified for the propylene glycol ratio (R^2^ = 0.99), good correlation for the water ratio (R^2^ = 0.82), and a moderate one for choline chloride (R^2^ = 0.58). A large difference stands out between the systems with a high degree of hydrogen bonding (systems 5, 24, and 23)—more than five bonds—and the systems with a low degree of hydrogen bonding (systems 6 and 22)—less than three bonds.

The systems comprised of mixtures of choline chloride, *β*-D-glucose, and water seem to have no connection between the hydrogen bonding and the ratio of the components (all R^2^ < 0.42). The observation can be interpreted to suggest that hydrogen bonding may not be the driving mechanism in the interaction of chlorogenic acid with the mixture of compounds as the eutectic solvent, but as a complementary one.

In the mixtures comprised by choline chloride, lactic acid, and water (systems 10–13, 29, and 31) the intermolecular hydrogen bonding between chlorogenic acid and the mixture seems to be strongly correlated with the ratio of choline chloride (R^2^ = 0.93), less to the ratio of lactic acid (R^2^ = 0.44), and totally independent to the ratio of water (R^2^ = 0.06).

**Figure 2:**
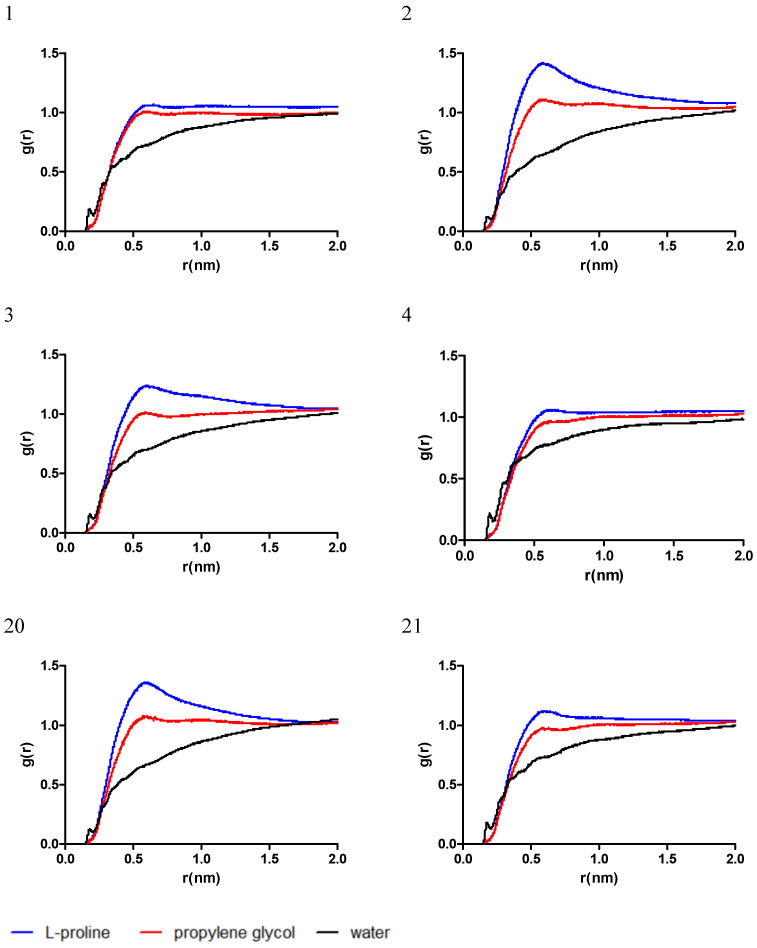
Radial distribution functions near chlorogenic acid in the mixtures comprised by L-proline as the HBA, propylene glycol as the HBD, and water for systems 1, 2, 3, 4, 20, and 21.

To gain a deeper understanding of the interactive behavior of the mixtures with the chlorogenic acid, the radial distribution function (RDF) was computed, as a measure of the probability of finding a molecule at a distance equal to the radial distance (r) from the chlorogenic acid considered as the reference molecule. The data for RDF are presented in plots in Figure 2.

Near the chlorogenic acid molecule, the positioning of the L-proline and propylene glycol is found to be dependent on the ratio of the respective molecules. In the mixtures 1, 4, and 21, which have the highest ratio of L-proline, there was a small difference between the g(r) for L-proline and propylene glycol, while in the mixtures 2, 3, and 20 a maximum g(r) was identified for L-proline near 0.5 nm, when compared to the g(r) of propylene glycol. On the other hand, the g(r) of water follows a similar trend in all cases, with a peak near 0.2 nm, corresponding to the formation of a hydrogen bond with chlorogenic acid.

**Figure 3:**
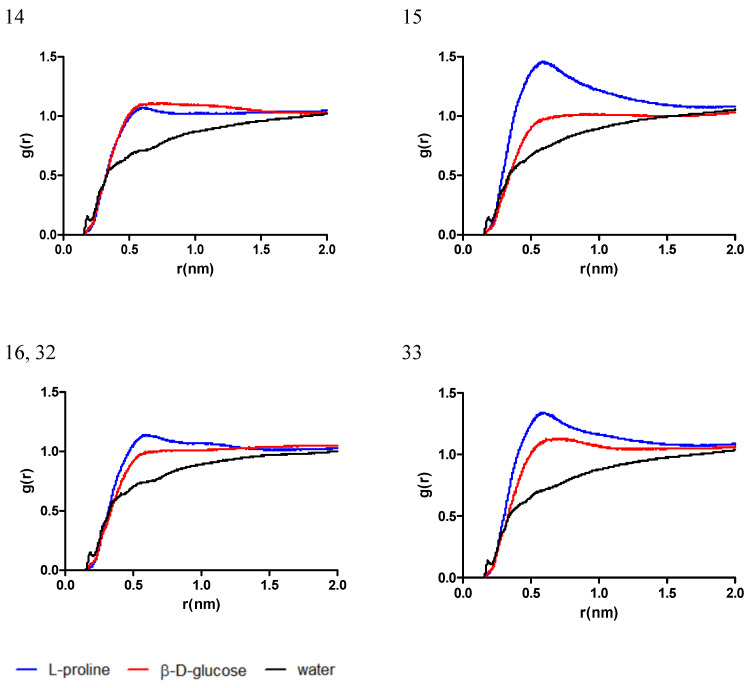
Radial distribution functions near chlorogenic acid in the mixtures comprised by L-proline as the HBA, *β*-D-glucose as the HBD, and water for systems 14, 15, 16, 32, and 33.

In the mixtures comprised of L-proline and β-D-glucose (systems 14, 15, 16, 32, and 33), the distribution of eutectic mixture molecules near chlorogenic acid have a similar trend as for system 14, comprised by the highest ratio of L-proline, while the respective ratio decreases in the order 16 = 32 > 33 > 15 and the difference in the g(r) of L-proline increases when compared to the g(r) of *β*-D-glucose. The g(r) of water follows a similar trend in all cases, with a peak near 0.2 nm, corresponding to the formation of a hydrogen bond with chlorogenic acid, as depicted in Figure 3.

**Figure 4:**
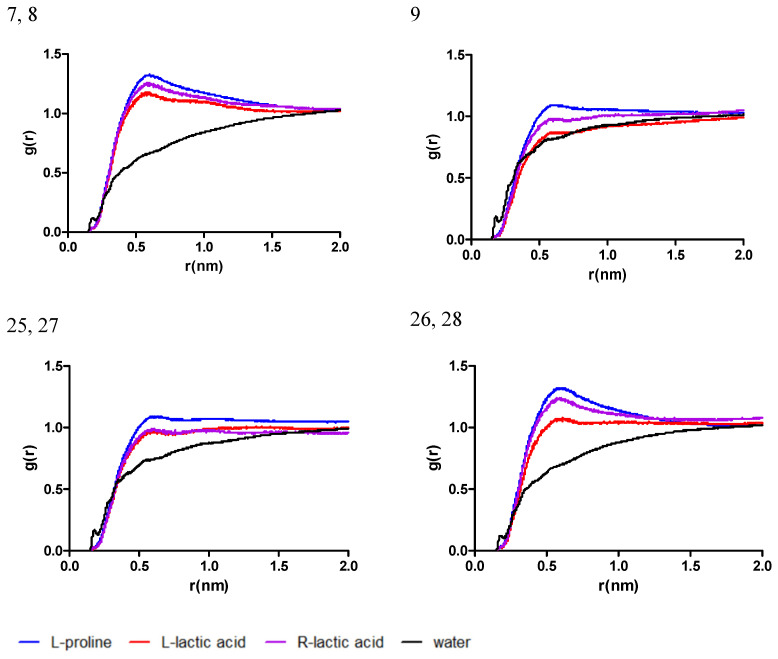
Radial distribution functions near chlorogenic acid in the mixtures composed of L-proline as the HBA, lactic acid as the HBD, and water for systems 7, 8, 9, 25, 26, 27, and 28.

The observations mentioned above are similar for simulated systems composed of mixtures of L-proline and lactic acid (systems 7–9, 25–28) and are shown in Figure 4. An increase in the L-proline ratio would lead to a similar trend of g(r) for L-proline and lactic acid and no significant peak in g(r) for systems 9, 25, and 27. On the other hand, for the systems with a lower L-proline ratio (systems 7, 8, 26, and 28), a prominent peak for g(r) can be identified near 0.5 nm, indicating an agglomeration of molecules near the chlorogenic acid molecule and the formation of a solvation shell nearby.

**Figure 5:**
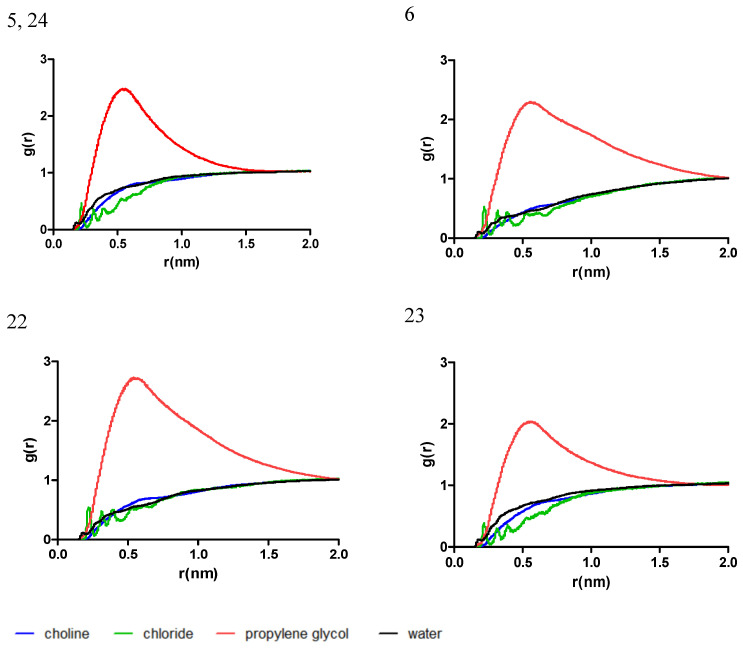
Radial distribution functions near chlorogenic acid in the mixtures composed of choline chloride as the HBA, propylene glycol as the HBD, and water in systems 5, 6, 22, 23, and 24.

In the mixtures 5, 6, and 22–24, where choline chloride was used as the HBA and propylene glycol as the HBD, it was identified that propylene glycol has the tendency to accumulate near the chlorogenic acid molecule; the plotting in Figure 5 indicates a high g(r) and a significant skewness, especially for the ones with a high propylene glycol ratio (particularly for systems 6 and 22). An interesting observation is regarding the g(r) for the chloride ion. The irregularities in its distribution may indicate a layered distribution of the molecules near the chlorogenic acid molecule.

**Figure 6:**
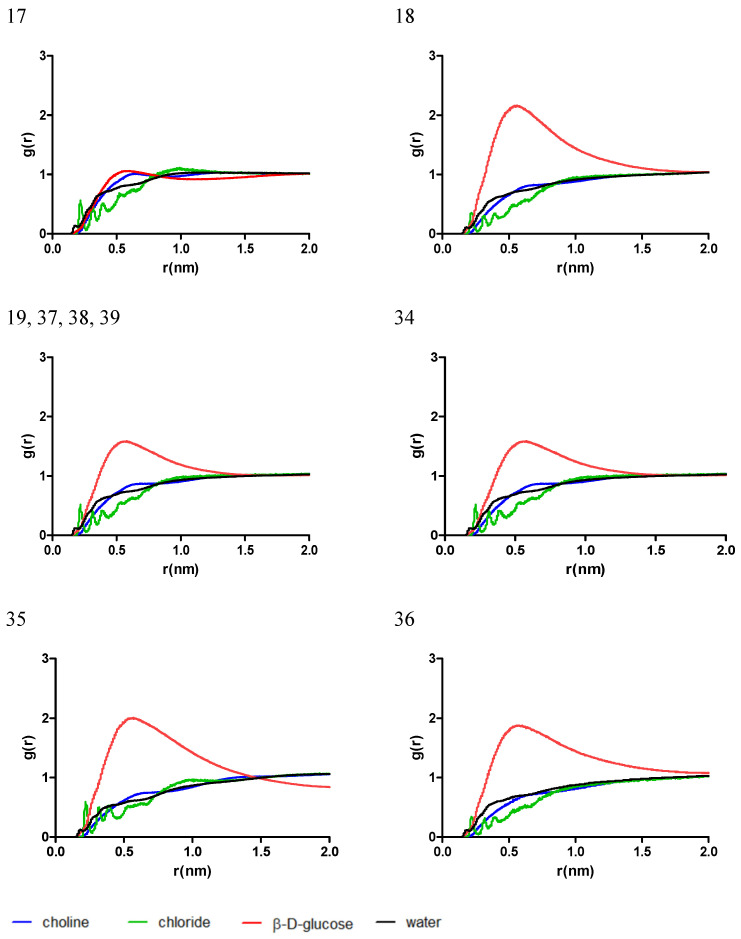
Radial distribution functions near chlorogenic acid in the mixtures comprised by choline chloride as the HBA, *β*-D-glucose as the HBD, and water for systems 17, 18, 19, 34, 35, 36, 37, 38, and 39.

In systems 17–19 and 34–39, comprised of choline chloride and *β*-D-glucose, the solvation of chlorogenic acid seems to be dependent on the ratio of *β*-D-glucose, as can be observed in Figure 6. In the mixtures with a moderate-to-low ratio of *β*-D-glucose (systems 18, 19, and 34–39), a significant increase in g(r) for *β*-D-glucose can be identified at 0.5 nm near chlorogenic acid. When the concentration of *β*-D-glucose increases (system 17), the distribution of *β*-D-glucose and choline chloride near chlorogenic acid appears similar, indicating a lower interaction with the solute model.

**Figure 7:**
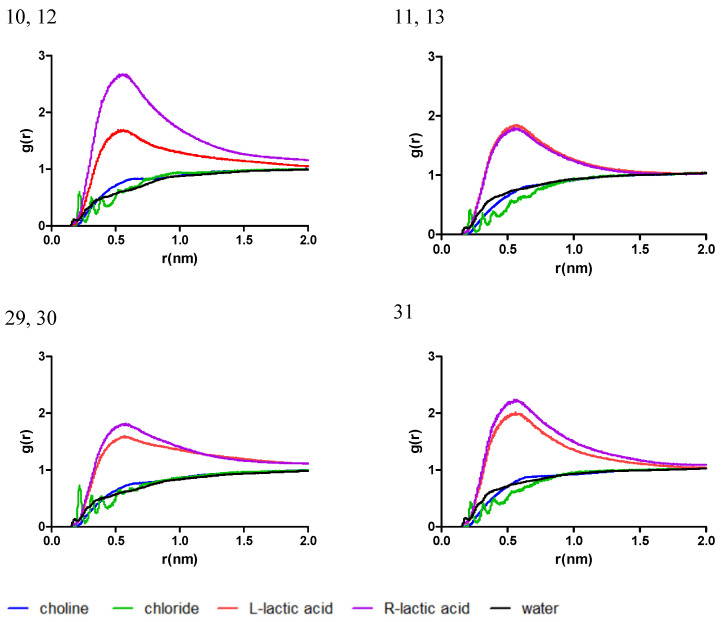
Radial distribution functions near chlorogenic acid in the mixtures comprised by choline chloride as the HBA, lactic acid as the HBD, and water for systems 10, 11, 12, 13, 29, 30, and 31.

As shown in Figure 7, in the mixtures consisting of choline chloride and lactic acid, similar to previous cases with choline chloride as the HBA, the interaction of the chlorogenic acid molecule with the mixture is mainly driven by the HBD. Interestingly, at high ratios of lactic acid a significant difference in g(r) for the two can be identified. R-lactic acid is much more present near chlorogenic acid when compared to L-lactic acid, the difference between them diminishing as their amount in the mixture decreases.

To better evaluate the interaction between chlorogenic acid and extraction mixtures, a non-bonding interaction analysis was performed to assess the solvation effects, and the resulting interaction energies are presented in Table 11 and Table 12.

Overall, it can be noticed that depending on the type of HBA, chlorogenic acid has a higher average electrostatic interaction in the systems using choline chloride as the HBD (−263.44 kJ/mol), than in the systems using L-proline as the HBD (−250.12 kJ/mol). Unsurprisingly, due to the chemical differences between the two types of HBD evaluated, in the studied systems the average van der Waals interaction of chlorogenic acid is higher with L-proline (−130.15 kJ/mol) than with choline chloride (−115.59 kJ/mol).

A more detailed analysis of the energetic interactions of chlorogenic acid with different HBD types reveals that *β*-D-glucose should be preferred, since it has the best interaction energies regardless of the type of HBA used (more than -390 kJ/mol on average). On the next level of interaction are the mixtures using lactic acid as the HBD, some of which reached high interaction levels with chlorogenic acid. Propylene glycol seems to be unfavorable to the solvation of chlorogenic acid, regardless of the ratio in our simulation or the HBA used.

### 2.5. Results of Antimicrobial Activity Evaluation

#### 2.5.1. Antimicrobial Activity—In Vitro Qualitative Study

Using the disk diffusion test, the initial screening technique was designed to identify the antimicrobial potential against some standard microbial strains. The results obtained for the selected blank NaDES mixtures and the corresponding extracts (those with content of chlorogenic acid higher than 1000 μg/g dw) and the extract obtained under optimal conditions are summarized in Table 13.

Overall, results regarding the antimicrobial activity of eutectic solvents (24–39) showed moderate antibacterial activity and less antifungal activity, but for certain solvents only, namely 25–30, except compound 39, which showed a weak antifungal activity against the *Candida albicans* strain (7.67 mm inhibition zone). The diameter of the inhibition areas for Gram-positive bacterial strains ranged from 6.75 to 23.68 mm. Overall increased efficiency was observed for solvents tested against the *Streptococcus pyogenes* strain, followed by *Staphylococcus aureus* and *Enterococcus faecalis.*

Regarding Gram-negative species, an overall moderate antimicrobial effect was noted, with inhibition zone diameters ranging from 9.26 to 19.03 mm, demonstrating a higher antibacterial potential against Gram-negative compared to the Gram-positive group. Within the Gram-negative strains, NaDESs showed increased activity against *Pseudomonas aeruginosa*, whereas for *Escherichia coli* and *Klebsiella pneumoniae* similar results were observed.

For NaDESs 24 and 31–39, no antibacterial effect was observed.

All plant extracts (24–39) that were screened for antimicrobial activity by the disk diffusion method showed an antimicrobial effect. More precisely, an increased efficiency was demonstrated against Gram-positive bacteria, with the diameters of growth inhibition areas ranging from 6.38 to 21.87 mm. Overall, tested extracts showed the highest antibacterial potential against *Streptococcus pyogenes*, followed by *Listeria monocytogenes*, *Staphylococcus aureus*, and *Enterococcus faecalis*. This time, antimicrobial potential was observed for extract 24, with a slightly increased efficiency for Gram-positive strains compared to Gram-negative ones, where the inhibition area was only present for *Klebsiella pneumoniae* (9.63 mm). However, for this extract in particular, the highest potential was demonstrated against the *Candida albicans* strain (13.69 mm).

Regarding Gram-negative bacteria, not all tested extracts showed an antibacterial effect against these strains. Therefore, only half the extracts demonstrated moderate efficiency, with extracts 32–39 showing no activity against *Escherichia coli* and *Klebsiella pneumoniae*. However, more than half of the extracts proved to have increased antimicrobial activity against the *Pseudomonas aeruginosa* strain.

In addition to the antibacterial activity, the tested plant extracts demonstrated an increased efficiency against the reference fungal strain, except extracts 26–28, 31, and 39. Inhibition areas diameters ranged from 8.86 to 28.04 mm. Hereby, a notable antifungal activity was observed for extracts 32-38, showing large diameters of the inhibition zones.

In addition, it is important to note that the influence of the NaDES mixtures used alone and the effect of the bioactive compounds from blackcurrant leaves are not completely separate. The results suggest that there may be potentiating effects between these two types of components, making it difficult to separate their individual contributions to antimicrobial activity.

#### 2.5.2. Antimicrobial Activity—In Vitro Quantitative Study

The disk diffusion test was used to select the NaDES mixtures and vegetal extracts with increased antimicrobial activity. The decision was to select the extracts and NaDESs that had inhibition areas higher than 10 mm, plus several others with values close to 10 or 0, to evidence the results of the disk diffusion test. The results from the qualitative study were confirmed by the quantitative assessment and they are summarized in Table 14.

The results regarding the antimicrobial activity of NaDESs demonstrated moderate antibacterial and antifungal activity. For Gram-positive bacterial strains, the minimum inhibitory concentration (MIC) ranged from 1/8 to 1/128, and minimum bactericidal concentration (MBC) from 1/2 to 1/64, with most NaDESs showing a bactericidal effect. Overall increased efficiency was observed for NaDESs tested against the *Listeria monocytogenes* strain, followed by *Streptococcus pyogenes*, *Staphylococcus aureus*, and *Enterococcus faecalis*.

Regarding Gram-negative species, the NaDES mixtures demonstrated an overall moderate antimicrobial effect with MIC and MBC values similar to the Gram-positive group. Within the Gram-negative strains, NaDESs showed increased activity against *Pseudomonas aeruginosa*, whereas for *Escherichia coli* and *Klebsiella pneumoniae* similar mean results were observed.

For *Candida albicans*, the antimicrobial potential of tested NaDESs was reduced, with most MIC values ranging from 1/4 and 1/8.

All plant extracts (24–39) were selected for the evaluation of antimicrobial activity against Gram-positive bacteria: 24–31 plus 35 against Gram-negative bacteria and 29–38 against the yeast strain. For Gram-positive bacteria, the tested extracts showed increased antibacterial potential against *Streptococcus pyogenes*, with very low values up to 1/2048, with a bactericidal MIC index for most extracts, followed by *Listeria monocytogenes*, *Enterococcus faecalis*, and *Staphylococcus aureus*. MIC index ≤ 4 was noted for extracts 25–30 against *Staphylococcus aureus* and *Enterococcus faecalis*. For the other extracts and two Gram-positive bacteria, the effect was mostly bacteriostatic.

The results for Gram-negative bacteria demonstrated increased efficiency, especially against *Pseudomonas aeruginosa*, where for extracts 25 and 26, but also 28–30 and 35–38, the MIC was 1/256 and 1/128. Moreover, all tested extracts against *Pseudomonas aeruginosa* had a bactericidal effect, with a MIC index of 1 or 2. Against both *Escherichia coli* and *Klebsiella pneumoniae*, extracts 25–30 demonstrated low MIC values, from 1/32 to 1/128, and a bactericidal MIC index.

Antimicrobial activity against the fungal strain resulted in low MIC values associated with extracts 35-38 (1/128 to 1/512), but, concerning the MIC index, they only demonstrated a fungistatic effect.

**Figure 8:**
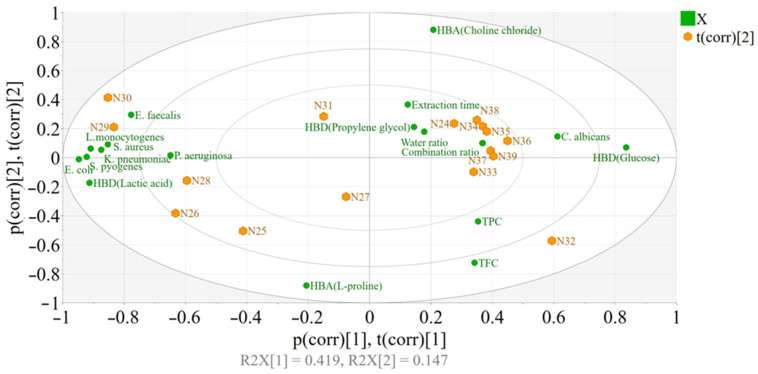
Bi-plot graph of the PCA model developed to evaluate the inhibitory effect on the growth of microorganisms (where E. faecalis—*Enterococcus faecalis*; L. monocytogenes—*Listeria monocytogenes*; S. aureus—*Staphylococcus aureus*; P. aeruginosa—*Pseudomonas aeruginosa*; K. pneumoniae—*Klebsiella pneumoniae*; S. pyogenes—*Streptococcus pyogenes*; E. coli—*Escherichia coli*; C. albicans—*Candida albicans*; HBD—hydrogen-bond donor; HBA—hydrogen-bond acceptor; TPC—total phenolic content, expressed as milligrams of gallic acid equivalents per gram of dry weight (mg GAE/g dw); TFC—total flavonoid content, expressed as milligrams of quercetin equivalents per gram of dry weight (mg QE/g dw)).

The experimental outcomes on the antimicrobial activity of NaDES extracts were analyzed using Principal Component Analysis (PCA) and are depicted in Figure 8. This analysis, in addition to providing a better overview of the dataset, underscores the varying effects of different factors on microbial inhibition and highlights specific correlations between these factors and response variables across microorganism groups. The results indicate a clear distinction between responses to *Candida albicans* and the other microorganisms investigated. The following input factors were found to have a minimal impact on the evaluated responses, as indicated by their central positioning in the PCA graph: extraction time, HBD type (namely propylene glycol), water ratio, and molar combination ratio (HBA:HBD). Notably, the type of HBD played a significant role: lactic acid (HBD) was associated with higher response values for non-*Candida* microorganisms, while *β*-D-glucose (HBD) correlated with increased response values for *Candida albicans*. The effect of the HBA was found to be negligible. Additionally, higher levels of TPC and TFC were correlated with improved responses against *Candida albicans*.

It is important to emphasize that antimicrobial activity is driven by the bioactive compounds extracted under specific conditions. While these conditions influence the extraction of these compounds, the more significant relationship lies between the compounds themselves and their antimicrobial effects rather than between extraction conditions and effects. To investigate this, we applied OPLS-DA models, which enabled us to assess the correlation between phytochemical compounds and antimicrobial activity (additional information is presented in the Supplementary Material and supported by Figure S1 and Table S2, respectively).

The antimicrobial data were not included in the experimental design because some extracts and NaDES blanks exhibited no antimicrobial activity. The absence of measurable results (0) in several cases made it unfeasible to model these data using the experimental design approach. However, following additional analysis with OPLS-DA models (presented in the Supplementary Material), antimicrobial activity was included in the final optimization phase. During this step, the optimal extraction conditions aligned with those previously established for maximizing TPC and TAA, as presented in Section 2.1.3.

## 3. Discussion

### 3.1. Quantitative Determination of Total Bioactive Compounds and Antioxidant Activity

The results of this research on *Ribes nigrum* (RN) leaf extracts prepared in different NaDES mixtures provide interesting information about the phytochemical profile of RN leaves harvested from the Obcinile Bucovinei, Suceava County, from the spontaneous flora of Romania at an altitude of 1000 m. In addition, the extensive study carried out for the optimization of extraction conditions (Table 1) highlights the high variability in the results and emphasizes the major impact of the experimental conditions on the content of bioactive compounds and antioxidant capacity of the extracts.

We mention that this is the first study of polyphenolic compounds extracted from RN leaves with NaDESs. There are no similar data reported in the literature for this plant species. Therefore, the comparison of the values obtained in the present research was made with the data reported for extracts prepared in classical solvents from this plant matrix (Supplementary Table S3).

Comparing the results obtained for TPC, the high capacity of some NaDESs to extract polyphenolic compounds is remarkable. The highest value recorded was 490.53 ± 0.10 mg GAE/g dw (sample N37—UAE, NaDES: ChChl:Glu, molar ratio 1.5:1, 40% water, 7.5 min) (Table 1), much higher than other values reported in the literature for TPC in RN leaves (Table S3). Babayan and Sahakyan found 167.15 ± 7.29 mg GAE/g dw in ethanolic extracts obtained from RN leaves collected from the wild flora of Armenia, at a 1600–1650 m altitude [36]. The values determined for RN from crops are significantly lower: Vagiri et al. reported 89–97 mg GAE/g dw for ethanolic extracts prepared by UAE [15], Stevic et al. found 40.1 ± 2.1 mg GAE/g dw in the variety Čačanska crna grown at 1000 m in Serbia [37], and Paunovic et al. obtained 2.17 mg GAE/g dw for the variety Titania grown at 242 m in Serbia [38], as well as Nowak et al. who also found 2.17 mg GAE/g dw in RN grown in Poland [39]. In some cases, comparison with literature data is difficult due to the different ways of expressing the results. Thus, Tabart et al. obtained a value of 46.0 ± 8.4 mg CAE/g frozen weight (fw) for polyphenols extracted from leaves of blackcurrant Noir de Bourgogne variety grown in Belgium by simple extraction for 1 h at 4 °C, but expressed the result in chlorogenic acid equivalents (CAE) per gram frozen weight (fw) [2]. Another example is that of Magnavacca et al. who analyzed leaves of RN cultivated in Italy and expressed the TPC value per g extract, obtaining 17.260 ± 0.473 mg GAE/g extract [40].

In terms of TFC, the extracts in NaDESs had a lower content, the highest value determined being 14.55 ± 0.04 (sample N32—UAE, NaDES: L-Pro:Glu, 1:1 molar ratio, 30% water, 5 min). In the wild variety of Armenian RN, the TFC values reported for ethanolic extracts obtained by classical extraction are three- or even four-times higher: 35.42 ± 1.52 mg QE/g dw [41] and 49.99 ± 0.86 mg QE/g dw [36], respectively. For cultivated RN cultivars, values are difficult to compare due to the different way of expressing the results: 2.05 ± 0.34 mg QE/g dw for the variety Noir de Bourgogne grown in Belgium [2] or 1.36 mg rutin equivalents (RuE)/g dw for the variety Titania grown at 242 m in Serbia [38].

There are several studies that have highlighted the high antioxidant potential of RN leaves, usually correlated with polyphenol content [41,42,43]. In a study by Hovhannisyan et al., all compounds in an extract from RN leaves were chromatographically separated and derivatized post-chloro-column with 2,2′-azino-bis(3-ethylbenzothiazoline-6-sulfonic acid (ABTS). The antioxidant activity of all separated and identified compounds was noted. The highest antioxidant activity was observed for flavan-3-ols, followed by hydroxycinnamic derivatives and flavonols [41]. In the DPPH assay, this extract was able to scavenge 50% of the free radicals at the concentration of around 63.59 mg/mL [41]. Similar values were obtained for extracts prepared from wild Armenian RN used in different studies and characterized for antioxidant activity by a DPPH assay: IC_50_ = 66.01 ± 1.65 μg/mL vs. positive control (catechin) = 13.08 μg/mL [36]. Okmen et al. determined the antioxidant activity of ethanolic, methanolic, and aqueous extracts obtained from RN leaves (150 mg/mL) in a Soxhlet apparatus. The DPPH free radical scavenging capacity was 29.5%, 83.3%, and 63.8%, respectively [43]. Teleszko and Wojdylo evaluated the antioxidant activity of leaf extracts for three varieties of RN by ABTS and FRAP assays and revealed their increased antioxidant potential correlated with high content of flavonols (mono-, di-, and oligomeric) and proanthocyanidins [42]. Soobrattee et al. categorized flavonoid compounds according to their antioxidant activity in the following order: dimer procyanidin > flavan-3-ols > flavonols > hydroxycinnamic acids > simple phenolic acids [32,44]. In our study, the highest antioxidant activity was recorded for sample N36: 263.13 ± 0.01 mg TE/g dw (UAE, NaDES: ChChl:Glu, molar ratio 1:1, 50% water, 10 min), a value close to that of the sample with the highest TPC content (N37) of 490.53 ± 0.10 mg TE/g dw (Table 1). The determination of flavanol content was performed only by LC-ESI-MS/MS and HPLC-DAD, not spectrophotometrically. As shown by the results presented in Table 7, the extracts prepared in NaDESs have a high flavanols content, which contributes significantly to antioxidant activity and free radical scavenging. Therefore, even though the TFC content is relatively low in the extracts in NaDESs, the high antioxidant potential is noticed, which motivates further investigation for their benefits. The reactivity variations observed between extracts in NaDESs in the DPPH assay may be attributed to variations in their polyphenolic composition, including compounds containing dihydroxyphenyl moieties, compounds with different molecular sizes (e.g., flavanols), other unidentified substances exhibiting antioxidant activity [32], or influences exerted by NaDES components on free radical scavenging reactions [45]. The solvent may modulate the antioxidant activity of polyphenols, even though their stability and structure remain unaffected. These effects require further studies and could present an interesting direction for further investigation.

### 3.2. Phytochemical Analysis of Ribes nigrum Leaf Extracts

Phytochemical analysis of RN leaves harvested from Romania’s wild flora allowed the identification and quantification of 26 phenolic compounds distributed in two major classes, phenolic acids and flavonoids, flavanols, and flavonols, respectively (Table 6, Table 7 and Table 8). Teleszko and Wojdylo also identified phenolic acids, flavanols, and flavonols as the main classes of polyphenolic compounds in blackcurrant leaf extracts. But they found that over 50% of the polyphenols contained in blackcurrant leaves were flavonols [42].

In our study, phenolic acids were the most abundant compounds in extracts prepared using NaDESs and UAE. Chlorogenic acid reached the highest concentration of 3992.21 µg/g dw in sample N24, which was extracted using a mixture of choline chloride and propylene glycol (molar ratio 2:1) with 50% water and a 10-min extraction time. This concentration is significantly higher than those reported in other studies, which found 20.9 ± 1.2 mg/g dw in methanolic extracts [46] and 35 ± 3 mg/g dw in hydroalcoholic extracts [47]. Additionally, previous studies reported chlorogenic acid concentrations in similar extracts of 1.5% of total extract weight (wt.%) [40] and 1.15% (wt%), respectively [5]. Nowak et al. detected the presence of chlorogenic acid only in trace amounts in aqueous extracts [39]. In another study, ethanolic extracts of RN leaves had chlorogenic acid amounts between 81 and 121 g/g dry leaves, depending on the position of the leaves on the branch and the date of harvest [15]. In another study performed by Tabart et al. (Table S3), gallic acid was the major phenolic acid in extracts obtained from RN leaves in acetone or after specific extraction: 1015 ± 54 μg/g fw, respectively 1883 ± 90 μg/g fw, followed by gentisic acid: about 1900 μg/g fw (in acetonic extract) or *p*-hydroxybenzoic acid: 1572 ± 32 μg/g fw (in case of specific extraction followed by hydrolysis) [2]. 

Flavanols have been identified in RN leaves systematically in phytochemistry studies [15,38,41,48]. Teleszko and Wojdylo found 429.95 mg/100 g dw (between 792.09 and 238.95 mg/100 g dw) polymeric proanthocyanidins and 194.79 mg/100 g dw (between 179.01 and 204.65 mg/100 g dw) mono-, di-, and oligomeric flavan-3-ols in methanolic extracts prepared by UAE from three different RN cultivars [42]. Gallocatechin, epigallocatechin, catechin, and epicatechin were quantified by Tabart et al. in acetone extracts, the first two reaching concentrations of 382 ± 132 μg/g fw and 150 ± 86 μg/g fw, respectively [2]. These compounds were also detected in the leaves of Estonian [49,50] and Armenian [41,48] RN (Table S3). Thus, blackcurrant is a significant source of condensed tannins.

In our study, NaDESs were able to extract epigallocatechin (maximum 43.03 μg/g dw), followed by epicatechin (maximum 36.58 μg/g dw), and catechin (maximum 11.92 μg/g dw) from the leaves of RN, and EGCG only in trace amounts. In contrast, procyanidins were efficiently extracted by UAE, seven procyanidins could be quantified. Sample N24 (UAE, NaDES: ChChl:PGL, molar ratio 2:1, 50% water, 10 min) contained the highest concentrations of C1 (217.76 μg/g dw) and B2 (150.05 μg/g dw) procyanidins (Table 7).

Another important group of polyphenols detected in RN leaves are flavonols, bioactive compounds with a strong antioxidant capacity. Stevic et al. found quercetin (84 ± 2.4 mg/g dw), kaempferol (43.6 ± 1.6 mg/g dw), and myricetin (9.5 ± 0.4 mg/g dw) in leaves of RN from Serbia (grown at 1000 m) after extraction in methanol and hydrolysis [37]. Tabart et al. also reported the presence of these three flavonolic aglycones in acetonic extracts of RN leaves in approximately the same proportion: quercetin (778 ± 203 μg/g fw), kaempferol (322 ± 151 μg/g fw), myricetin (139 ± 47 μg/g fw) [2]. Paunovic et al. found myricetin (0.241 ± 0.02 mg/100 g dw) in higher amounts than kaempferol (0.116 ± 0.01 mg/100 g dw), but also quercetin present in higher concentrations (0.712 ± 0.05 mg/100 g dw) in ethanolic extracts prepared by UAE [38].

Flavonols found in plants are often in glycosylated form. Several quercetin glycosides have been identified in RN leaves: hyperoside (quercetin-3-*O*-galactoside), isoquercetin (quercetin-3-*O*-glucoside), rutin (quercetin-3-*O*-rutinoside), quercitrin (quercetin-3-*O*-ramnoside), but also quercetin-3-*O*-glucoronide, quercetin acetyl glucoside, quercetin-3-(6″-malonyl)-glucoside, quercetin-3-*O*-glucosyl-6″-acetate, rhamnetin glucoside, isorhamnetin-3-*O*-rutinoside, isorhamnetin-3-*O*-glucoside, and others [5,15,47,48,50]. Heterosides of kaempferol (kaempferol-3-*O*-rutinoside, kaempferol-3-*O*-glucoside, kaempferol galactoside, kaempferol-malonyl-glucoside, etc.) were detected in significantly lower amounts [5,15,39,47].

The extracts prepared from RN leaves in NaDESs and analyzed in this study mainly contained quercetin heterosides: isoquercetin (maximum 466.39 μg/g dw), quercitrin (maximum 230.85 μg/g dw), hyperoside (maximum 216.59 μg/g dw), and rutin (maximum 126.01 μg/g dw), with quercetin, kaempferol, and kaempferol-3-*O*-rhamnoside present in trace amounts (Table 8). The levels of flavonols in the extracts vary quite widely depending on the extraction conditions.

UTE is a modern and environmentally friendly extraction method, less commonly applied on plant matrices. It has been efficiently used by our research team for the extraction of bioactive compounds, including polyphenolic compounds from walnut septum [51,52], hazelnut involucre [53], and more recently from RN young shoots [32], with better results compared to UAE. In the present study, in the presence of NaDESs, UTE was more efficient than UAE only in extracting some flavonols (especially isoquercetin and rutin) from RN leaves (Table 8).

With their high polyphenolic content, varied phytochemical profile, and demonstrated antioxidant activity, extracts obtained in NaDESs from RN leaves collected from the mountainous area in the extreme north region of Romania are of interest for biological investigations.

### 3.3. Investigation of Antimicrobial Activity for Ribes nigrum Leaf Extracts

Following the evaluation of the antimicrobial activity of the tested extracts, certain trends became clear. Extracts N25–N30 and N35–N38 demonstrated notable efficacy against the tested microorganisms, as evidenced by both the diameters of the inhibition zones and the MIC values obtained. These results highlight a remarkable therapeutic potential, particularly against *Streptococcus pyogenes* and *Pseudomonas aeruginosa*, for which the lowest MIC values were recorded. Additionally, significant antifungal activity was observed for extracts N32-N38, suggesting their potential use in combating fungal infections caused by *Candida albicans*.

When comparing the results from diffusion and dilution tests, it was observed that the extracts exhibited a bactericidal effect against certain Gram-positive and Gram-negative strains, further confirming their potential as antimicrobial agents. However, it is important to note that the antifungal activity observed against *Candida albicans* was predominantly fungistatic.

In addition to the results obtained for the tested extracts, it is important to note that the extract prepared under the optimal conditions identified through the experimental design (OE) was validated. The antimicrobial activity of this extract was consistent with the predicted values, further confirming its efficacy.

Stevic et al. demonstrated that the essential oil of the RN leaves has in vitro antimicrobial activity against some bacterial strains and *Candida albicans*, with MIC values ranging between 1.0 and 27.0 μL/mL [37]. The most effective results were against the *Trichophyton mentagrophytes* isolate (MIC = 1.0 μL/mL), followed by the bacteria *Escherichia coli*, *Streptococcus faecalis*, and *Staphylococcus aureus* (MIC = 2.7 μL/mL), as well as against *Candida albicans* (MIC = 2.7 μL/mL). In our study, RN leaf extracts prepared in NaDESs showed good efficacy against Gram-positive bacteria, especially against *Streptococcus pyogenes*, *Enterococcus faecalis*, and *Listeria monocytogenes*, but only moderate efficacy against Gram-negative bacteria (Table 14). In the case of *Candida albicans*, NaDES extracts demonstrated good activity, but they were predominantly fungistatic. The differences in the phytochemical profiles of NaDES extracts and the essential oil from RN leaves directly influence their antimicrobial activity. The essential oil is rich in volatile compounds like carene and β-caryophyllene, which provide strong antimicrobial activity against Gram-positive and Gram-negative bacteria, and against *Candida albicans*. In contrast, NaDES extracts, which are rich in polyphenols such as chlorogenic acid, epicatechin, and isoquercetin, are more effective against Gram-positive bacteria and exhibit predominantly fungistatic activity against *Candida albicans*. This suggests that the essential oil has a stronger effect on microorganisms due to its volatile compounds, while NaDES extracts offer more specific and diversified activity depending on their polyphenolic composition.

The extracts obtained from RN leaves showed antimicrobial activity in Raudsepp’s study, especially those prepared in 96% ethanol, with inhibition zones ranging from 7 to 14 mm for Gram-positive bacteria and 8 to 11 mm for Gram-negative bacteria [50]. Comparatively, our NaDES extracts seem to have similar or slightly lower activity in some cases but demonstrate stronger activity against *Staphylococcus aureus*.

In Hovhannisyan et al.‘s study, silver nanoparticles synthesized using an RN leaf extract were tested only against *Escherichia coli* strains, including antibiotic-resistant strains [41]. Although the leaf extract did not demonstrate antimicrobial activity, the silver nanoparticles showed significant antibacterial activity, with a reported MIC of 10 µg/mL. Comparing the antimicrobial activity of RN extracts prepared in NaDESs with silver nanoparticles synthesized using an RN leaf extract, we observed that each method offers distinct advantages. NaDES extracts present a broad antimicrobial spectrum, effective against both Gram-positive and Gram-negative bacteria, as well as fungi. In contrast, silver nanoparticles offer very effective activity against *Escherichia coli* strains, especially those resistant to antibiotics.

Paunović et al. reported MIC values ranging from 80.02 to 199.21 µg/mL for an RN leaf extract against bacteria and fungi [38]. The lowest MIC value of 80.02 µg/mL was obtained against *Candida albicans*, indicating significant antifungal activity. In our study, the NaDES extract 38 showed the best activity against *Candida albicans*, with a MIC value of 1/512 (Table 14).

Babayan et al. detected no antimicrobial activity for an ethanolic extract prepared from RN leaves tested against Gram-positive bacteria (*Bacillus subtilis*, *Staphylococcus aureus*, *Enterococcus hirae*), Gram-negative bacteria (*Escherichia coli*, *Salmonella typhimurium*), and two *Saccharomyces cerevisiae* strains [36].

Comparing the data from literature with the results of our study, the superior efficacy of the extracts obtained in NaDESs is evident, especially against Gram-positive and Gram-negative strains. These results suggest the considerable potential of these extracts in antimicrobial treatments.

To better understand this efficacy, it is essential to explore the antimicrobial mechanisms through which polyphenols exert their effects. Polyphenols from *Ribes nigrum* L. exhibit effective antimicrobial activity by disrupting bacterial cell membranes, increasing permeability, and leading to bacterial destruction [54]. This effect is particularly important for Gram-positive bacteria, which have a simpler membrane structure and are thus more susceptible to these disruptions.

Another mechanism by which polyphenols act is by inhibiting essential bacterial enzymes such as proteases and nucleases, thereby disrupting critical metabolic processes and blocking bacterial growth and replication. In the studies of Olivares et al., it was demonstrated that NaDESs can maintain the activity of antibiotics by stabilizing their chemical structure, thereby allowing the continuous inhibition of bacterial enzymes [54,55]. This stability also applies to polyphenols, maximizing their inhibitory effects on bacterial enzymes and amplifying the overall antimicrobial effect.

Additionally, polyphenols interfere with bacterial DNA and RNA syntheses, disrupting replication and transcription processes, which prevents bacterial proliferation and is essential in combating bacterial infections. Similarly, Olivares et al. reported a MIC value of 1 µg/mL against *Pseudomonas aeruginosa* for imipenem stabilized in a NaDES [54]. This value is comparable to those obtained in our study against *Pseudomonas aeruginosa*, underscoring the efficacy of the compounds analyzed by both studies in inhibiting this pathogenic bacterium.

Nystedt et al. observed that NaDESs not only protect polyphenols from degradation but also amplify their antimicrobial effects by contributing to the destabilization of bacterial membranes and inhibiting biofilm formation [56]. In their study, they tested three types of NaDESs to evaluate their ability to break down the biofilm matrix and reduce the viability of bacterial cells in preformed *Staphylococcus aureus* and *Pseudomonas aeruginosa* biofilms. The results showed that these NaDESs significantly reduced the number of viable cells by 4-6 orders of magnitude, and all three NaDESs inhibited biofilm formation, demonstrating their potential as anti-biofilm agents in future antimicrobial formulations. The results of our study are also supported by Bedair et al.’s study, which demonstrated the antimicrobial efficacy of NaDESs [57]. In this study, NaDESs generated significant inhibition zones and presented very low MIC values, indicating remarkable antimicrobial activity. The observed mechanisms of action include the destabilization of bacterial cell membranes and the disruption of osmotic balance, contributing to cell lysis. These effects are intensified by the acidic apparent pH of NaDESs, which helps to destroy essential proteins for microorganism survival. Similarly, polyphenols from RN act as antimicrobials by destabilizing membranes and disrupting nucleic acid synthesis, effects that become stronger in the presence of NaDESs. The use of NaDESs not only stabilizes polyphenols by preventing their degradation, but also enhances their antimicrobial mechanisms, ensuring effective and long-lasting activity against both Gram-positive and Gram-negative bacteria.

Thus, the synergy between NaDESs and polyphenols enhances antimicrobial activity and offers a promising alternative to traditional antimicrobial agents, especially in the context of increasing bacterial resistance.

### 3.4. Molecular Dynamics Simulations Outcomes

In the studied mixtures, it was identified that an excessive increase in the ratio of L-proline as the HBA would lead to diminishing the interaction between the eutectic mixture and chlorogenic acid taken as the solute model. On the other hand, based on the range tested, a large amount of choline chloride had no negative effect on the solvation of chlorogenic acid. R-lactic acid seems to be much more present in the solvation shell of chlorogenic acid than L-lactic acid. The results of the molecular dynamics studies indicated that the desired HBD should be *β*-D-glucose or lactic acid and not propylene glycol for the extraction of chlorogenic acid taken as the solute model.

However, to gain a clearer understanding of the behavior of the mixtures in molecular dynamics studies, it would be necessary to evaluate more systems, with step-by-step variation in only one parameter (for example, the ratio of a component), to obtain continuous results and to conclude which are the minimum and the maximum ratios of each component. The present in silico study was targeted to evaluate the systems used in extractions in the wet lab as discrete situations, not an extensive study on a large range of parameters.

## 4. Materials and Methods

### 4.1. Chemicals and Reagents

All reagents used in this study were of analytical purity. Sodium carbonate, 6-hydroxy-2,5,7,8-tetramethylchroman-2-carboxylic acid (Trolox) (97%), and 2,2-diphenyl-1-(2,4,6-trinitrophenyl) hydrazine (DPPH) were purchased from Sigma (Sigma Aldrich Chemie GmbH, Schnelldorf, Germany). Aluminum chloride (≥98%) was obtained from Carl Roth (Karlsruhe, Germany), while the Folin–Ciocâlteu reagent and the solvents: acetone, ethanol, and methanol, all HPLC purity, were supplied by Merck (Darmstadt, Germany).

The following standards used for both spectrophotometric and LC-MS analyses were obtained from Sigma Aldrich (Sigma Aldrich Chemie GmbH, Schnelldorf, Germany): epigallocatechin (EGC) and epigallocatechin gallate (EGCG) standards, apigenin, caffeic acid, 4-*O*-caffeoylquinic acid, caftaric acid, (+)-catechin, chlorogenic acid, p-coumaric acid, (−)-epicatechin, fisetin, gentisic acid, hyperoside (quercetin 3-D-galactoside), isoquercitrin (quercetin 3-β-D-glucoside), kaempferitrin, kaempferol, kaempferol-3-rhamnoside, luteolin, myricetin, patuletin, procyanidins A1, B1, B2, B3, B4, C1, and C2, protocatechuic acid, quercetin, quercitrin (quercetin 3-rhamnoside), rutin (quercetin-3-O-rutinoside), syringic acid, vanillic acid, vitexin (apigenin 8-C-glucoside), and vitexin 2-O-rhamnoside. Sinapic acid was purchased from Carl Roth (Karlsruhe, Germany), while ferulic acid and gallic acid were obtained from Merck (Darmstadt, Germany). HPLC-grade methanol and analytical-grade acetic acid were purchased from Merck KGaA (Darmstadt, Germany). Water of Milli-Q quality was used throughout the study.

### 4.2. Plant Samples

Blackcurrant (*Ribes nigrum* L.) leaves were collected from the Obcinile Bucovinei region, Suceava County, Romania (47°53′23′′ N, 25°17′21′′ E, approximately 1000 m altitude) in June 2022. A sample was identified at the Department of Pharmaceutical Botany and included in the herbarium of this department at the Faculty of Pharmacy, Iuliu Hațieganu University of Medicine and Pharmacy, Cluj-Napoca, Romania (registration number *Ribes nigrum* L.—51.1.2.1.1).

After harvesting, the plant material was cleaned by rinsing with water. The fresh leaves were dried at room temperature in the dark for 10 days and then chopped by cutting and grinding using a Thermomix device (Vorwerk, Germany). The plant material thus prepared was immediately used in the extractions performed within the experimental design.

### 4.3. Experimental Design

To optimize the extraction process of bioactive compounds from *Ribes nigrum* L. leaves, a D-optimal design was used using Modde Software, version 13.0 (Sartorius Stedim Data Analytics AB, Umeå, Sweden). Six independent variables were considered: two HBAs—L-proline and choline chloride (X_1_), three HBDs—lactic acid, glucose, and propylene glycol (X_2_), the molar ratio of HBA:HBD (X_3_), the percentage of water in the solvent (X_4_), the extraction method—UAE or UTE (X_5_), and the extraction time (X_6_). The dependent variables were the total phenolic content (TPC) expressed as milligrams of gallic acid equivalents per g dry weight (mg GAE/g dw), total flavonoid content (TFC) expressed as milligrams of quercetin equivalents per g dry weight (mg QE/g dw), and total antioxidant activity determined by the DPPH assay (TAA-DPPH) expressed as milligrams of Trolox equivalents per g dry weight (mg TE/g dw) (Table 15).

During the screening step, MLR (multiple linear regression) was used to estimate the effect of independent variables and their interactions by means of polynomial equations. R^2^ and Q^2^ were employed as statistical parameters. R^2^, which indicates the proportion of response variation explained by the model, may overestimate the goodness of fit. Conversely, Q^2^, reflecting the proportion of response variation predicted by the model through cross-validation, may underestimate it. High R^2^ and Q^2^ values suggest a model with strong predictive power, in conjunction with a high value of model validity. Additionally, the model’s reproducibility was assessed by comparing the variation in responses under identical experimental conditions (pure error) to the total response variation.

### 4.4. Preparation of Extracts

NaDES mixtures were first prepared according to the experimental design (Table 1) by mixing the HBA and HBD in the appropriate molar ratio and stirring at room temperature using a Vortex RX-3 (Velp Scientifica, Usmate, Italy).

Next, the weighed plant material (Ohaus Discovery Balance) was extracted with the appropriate NaDES in 50 mL Falcon tubes in two steps: (a) shaking for 2 min using Vortex RX-3; (b) extraction itself either by UAE in an ultrasonic bath (S-180H Elmasonic, produced by ELMA) or by UTE with an ultra-turrax homogenizer (T18; IKA Labortechnik, Staufen, Germany) (Table 2). After extraction, each mixture was centrifuged at 10,000 rpm for 15 min at room temperature using a Centrifuge 204 Sigma (Osterode am Harz, Germany). Supernatants were separated, analyzed by spectrophotometry to determine the dependent variables of experimental design—TPC, TFC, and TAA-DPPH (Table 1)—and by LC-MS for the analysis of individual polyphenols, respectively, and tested for antimicrobial activity. The extracts obtained were stored in a refrigerator at 4 °C until analysis.

### 4.5. Quantification of Total Bioactive Compounds

#### 4.5.1. Total Phenolic Content

TPC was determined in extracts using the classical spectrophotometric method with the Folin–Ciocâlteu reagent developed for microplates according to the protocol previously described [32,53]. Briefly, 20 μL of each sample was placed in a 96-well plate and successively mixed with 80 μL of FC reagent (diluted 1:10) and, after a 3 min incubation period, with 80 μL of a sodium carbonate solution (7.5% *w*/*v*). The resulting solution was incubated in the dark at room temperature for 30 min. The absorbance was measured at 760 nm against a reagent blank using a Synergy HT microplate reader (BioTek Instruments, Inc., Winooski, VT, USA). Gallic acid was the reference standard, and the calibration curve was generated for the concentration range of 10–100 µg/mL (R^2^ = 0.9975). The results were expressed as mg GAE/g dw. Each extract was analyzed in triplicate.

#### 4.5.2. Total Flavonoid Content

TFC was also determined in extracts by a spectrophotometric method developed for microplates, following the methodology described previously [32,51]. Briefly, 100 μL of each extract was mixed with 100 μL of 2% AlCl_3_ aqueous solution. The resulting solution was incubated in the dark at room temperature for 15 min. The absorbance was measured at 420 nm against a reagent blank, using the same microplate reader. Quercetin was the reference standard, and calibration curve was prepared in the range of 20–150 µM (R^2^ = 0.9937). The results were expressed as mg QE/g dw. Each extract was analyzed in triplicate.

### 4.6. Determination of the Antioxidant Activity by DPPH Assay

The antiradical capacity of the extracts by scavenging the free radical DPPH was measured using microplates [32]. For this purpose, 10 μL of each extract was mixed with 90 μL of a 0.004% methanolic solution of DPPH and incubated in the dark for 30 min. The absorbance was measured at 517 nm against a solvent blank using the Synergy HT microplate reader. The DPPH scavenging capacity was calculated in comparation with Trolox as the reference standard (good linearity for the concentration range of 1.34–67 µg/mL R^2^ = 0.9555 and the results were expressed as mg TE/g dw.

### 4.7. Phytochemical Analysis of Polyphenols by LC-MS

The phytochemical composition of the *Ribes nigrum* leaf extract was analyzed using liquid chromatography–tandem mass spectrometry (LC-MS/MS), employing two previously validated analytical methodologies [58]. The analysis was conducted using an Agilent Technologies 1100 Series HPLC system (Agilent, Santa Clara, CA, USA), equipped with an autosampler, column thermostat, binary gradient pump, degasser, and UV detector. This system was connected to an Agilent Ion Trap 1100 SL mass spectrometer (LC/MSD Ion Trap VL) [58,59].

In the initial analytical approach, chromatographic separation was achieved using a Zorbax SB-C18 reverse-phase column (100 mm × 3.0 mm i.d., 3.5 μm particle size, Agilent Technologies). The mobile phase involved a binary gradient of methanol and 0.1% acetic acid (*v*/*v*). The process began with 5% methanol, which gradually increased to 42% over 35 min, followed by an isocratic phase at 42% for 3 min. The column was then rebalanced with 5% methanol over the following 7 min [60]. The column was kept at 48 °C, with a flow rate of 1 mL/min and an injection volume of 5 μL. Detection utilized both UV and MS modes, with the UV detector set at 330 nm for polyphenolic acids for up to 17 min, and at 370 nm for flavonoids and their aglycones for up to 38 min. The MS system was operated in negative ESI mode, with a capillary voltage of +3000 V, a nebulizer pressure of 60 psi (using nitrogen), and a gas flow rate of 12 L/min at 360 °C [59,60].

Another validated LC-MS analytical method was applied to identify the possible presence of eight additional polyphenols: epicatechin, catechin, syringic acid, gallic acid, protocatechuic acid, vanillic acid, epigallocatechin, and epigallocatechin gallate. The same column and equipment settings were used, but the mobile phase was modified, starting at 3% methanol, increasing to 8% within 3 min, and reaching 20% at 8.5 min, maintained until 10 min, after which it returned to 3%. The detection of bioactive compounds was carried out in MS mode, with ESI parameters comparable to those used in the initial method [61,62].

To identify each bioactive compound, MS spectra and traces were compared with library standards, and quantification was performed using UV detection, based on the calibration curves of the relevant analytical standards. The data were analyzed and processed using Agilent’s DataAnalysis (v5.3) and ChemStation (vB01.03) software, with the results expressed in micrograms per mL of plant extract.

For the quantification of procyanidins in the vegetal extracts, a new LC-MS analytical method was developed, for which the same equipment used for polyphenolic compound analysis was employed. Chromatographic separation was achieved using a Zorbax SB-C18 column (100 mm × 3.0 mm i.d., 3.5 μm) from Agilent Technologies. A gradient of methanol and 0.1% acetic acid in water was used for separation, starting with 8% methanol, increasing to 20% at 8 min, followed by a 3 min re-equilibration at 8% methanol. The chromatography was conducted at 45 °C, with a flow rate of 1 mL/min and an injection volume of 5 μL. Analyte detection was performed using an ion trap mass spectrometer in MS/MS mode with negative ionization and an ESI source. The specific parameters included a capillary voltage of 3000 V, nebulizer pressure of 60 psi (nitrogen), a dry nitrogen gas flow rate of 12 L/min, and a dry gas temperature of 350 °C [32].

Under these conditions, the retention times for the procyanidins were the following: B3 at 2.6 min, B1 at 2.9 min, B4 at 3.8 min, B2 at 5.1 min, C1 at 7.1 min, and A1 at 7.6 min. For accurate quantification, the following mass spectrometry transitions were used: *m*/*z* 575 > (*m*/*z* 407; 423; 447; 449; 450; 453; 539; 557) for A1 procyanidin, *m*/*z* 577 > (*m*/*z* 407.2; 425.1; 451.1) for all four B procyanidins (due to their identical MS/MS spectra from their isomeric nature), and *m/z* 865 > (*m*/*z* 407.2; 425.2; 451.2; 543.3; 577.3; 695.4; 713.3; 739.3) for C1 procyanidin. The calibration curves for all analytes were linear across the concentration range of 0.1–100 μg/mL [32].

The results were processed using DataAnalysis (v5.3) and ChemStation (vB01.03) software from Agilent, with quantifications expressed as micrograms per gram of dry vegetal extract.

### 4.8. Methodology for Molecular Dynamics Simulations

To describe at a molecular scale the behavior over time of the evolution of the eutectic mixtures used as solvents in the extracts, a molecular dynamics simulation was carried out using GROMACS 2023 [63,64]. The force field used in the simulations was the Optimized Potentials for Liquid Simulations-All Atom (OPLS-AA) [65,66,67]. The structures of the molecules used in the simulations were optimized using Spartan20 (Wavefunction, CA, USA) on the B3LYP level of theory with the 6-311+G(d,p) basis set [68]. The parametrizations were performed using SwissParam [69,70]. The simulation systems were constructed by randomizing the position of components using PACKMOL in a 8 nm × 8 nm × 8 nm box [71]. The respective size was chosen as an intermediate value between the sizes of sides (lowest 5 nm and highest 10 nm) identified in the literature reports on the topic of the extraction of vegetal components using eutectic solvents [72,73,74,75]. In the center of each simulated system, one molecule of chlorogenic acid was placed, chosen as a model solute because its molecule has a complex structure which possesses various structural moieties such as three secondary alcohols, two phenols, and an α-β unsaturated ester connecting a cyclohexane ring to a benzene ring. Furthermore, significant variability in the quantification of chlorogenic acid was observed depending on the extraction mixture used. This variation highlights its potential interest for molecular dynamics studies. The cutoff radius was set to 1.2 nm for the non-bonded interactions, while the long-range ones were computed with PME [76]. The systems were equilibrated at the NPT and NVT ensembles for 5 ns and 50 ns, respectively. The production of simulation was performed for 100 ns at 300K and 1 bar. The number of molecules of solvent mixture in each box was determined according to the molar ratio of the components used in the extractions performed (HBA, HBD, and water), using a trial-and-error approach to ensure proper filling of the simulation box. Table 16 presents the composition of the systems using L-proline as the HBA is presented, while in Table 17 presents the composition of the systems using choline chloride as the HBA.

### 4.9. Antimicrobial Activity Assay Description

#### 4.9.1. In Vitro Qualitative Study

The antimicrobial potential was assessed using a two-step method. Initially, the disk diffusion test was used as a screening method to identify the extracts with high antimicrobial potential against reference strains of Gram-positive, Gram-negative bacteria, and yeasts. The microbial strains selected for the study were represented by four Gram-positive bacteria: *Staphylococcus aureus* ATCC 6538P, *Streptococcus pyogenes* ATCC 19615, *Listeria monocytogenes* ATCC 13932, *Enterococcus faecalis* ATCC 29212; three Gram-negative: *Escherichia coli* ATCC 13076, *Klebsiella pneumoniae* NCTC 13438, and *Pseudomonas aeruginosa* ATCC 27853; and one yeast strain: *Candida albicans* DSMZ 1386. Antibacterial and antifungal standard controls were also used in this study, namely Amoxicillin (10 µg, BioMaxima, Poland) for Gram-positive bacteria, Gentamicin (30 µg, BioMaxima, Poland) for Gram-negative strains, and Ketoconazole (10 µg, Liofilchem, Italy) for yeasts.

Screening was performed following the EUCAST standards [77], using an adapted disk diffusion method. Twenty-four-hour colonies, previously grown on Mueller–Hinton agar (MH, Merck, Germany) for bacteria and Sabouraud dextrose agar (SDA, Merck, Germany) for yeasts, were used to prepare the inoculum in sterile saline. The turbidity was adjusted to 0.5 density on the McFarland scale using a densitometer (DEN-1, Biosan, Latvia). The suspension was used to inoculate 8.5 cm-diameter plastic Petri dishes with MH agar for bacteria and SDA agar for yeast. A total amount of 10 µL was placed on filter paper discs (5 mm diameter), arranged in a radial model [61,77]. The plates were then incubated for 18 h at 35 ± 2 °C for bacteria and 48 h at 28 °C for the fungal strain antimicrobial activity was assessed by measuring the diameter of the growth inhibition zone, expressed in mm [61,77].

#### 4.9.2. In Vitro Quantitative Evaluation—MIC and MBC

The quantitative assessment was performed using the broth microdilution method to establish the minimum inhibitory concentration (MIC) and minimal bactericidal concentration (MBC) for all extracts tested against reference microbial strains. This evaluation was conducted according to the modified EUCAST protocols [78,79]. Briefly, the method was performed using 96-well titer “U”-shaped plates containing the extracts diluted in liquid MH medium and inoculated with 20 µL from the microbial suspension. The stock solutions of the extracts were diluted using a two-fold serial dilution system in consecutive wells, from the initial stock concentration (1/1) to the highest (1/2048). The final volume was adjusted to 200 µL (180 µL extract + 20 µL microbial inoculum). Positive and negative controls were represented by microbial inoculum in MH broth and uninoculated MH broth, respectively.

The plates were incubated for 24 h at 37 °C for bacteria and 48 h at 28 °C for the tested yeast. MIC values were determined as the lowest extract concentration able to inhibit visible microbial growth (with the same OD as the negative control), compared to the positive control, as established by a decreased value of absorbance at 450 nm (HiPo MPP-96, Biosan, Latvia) [59].

To establish MBC values, 10 µg of each well showing no visible growth was inoculated on MH agar and incubated for 24 h at 37 °C. MBC was therefore recorded as the lowest extract concentration killing 99.9% of the microbial inoculum after incubation [78,79].

In addition to MIC and MBC values, the MIC index was calculated to establish whether extracts possessed a bactericidal or bacteriostatic effect. The results were interpreted as bactericidal for an MBC/MIC ratio ≤ 4 or bacteriostatic for MBC/MIC > 4 [78]. All experiments were performed in triplicate.

## 5. Conclusions

This study demonstrated the effectiveness of using natural deep eutectic solvents (NaDESs) for the extraction of bioactive compounds from *Ribes nigrum* leaves. The optimization of extraction conditions resulted in extracts rich in polyphenols and flavonoids, with significant antioxidant activity. The results suggest that NaDESs represent a sustainable and efficient alternative to traditional organic solvents, improving the extraction yield. Additionally, antimicrobial tests confirmed the biological activity of these extracts against Gram-positive bacteria and *Candida albicans*, highlighting their potential for application in pharmaceutical and nutraceutical formulations. Given these promising results, further research is needed on other plant species to fully explore the potential of NaDESs in obtaining new therapeutic and antimicrobial agents.

## Figures and Tables

**Table 1:** Independent and dependent variables of the experimental design used to optimize the extraction process of bioactive compounds from blackcurrant leaves.

Exp. Name	Run Order	HBA (X_1_)	HBD (X_2_)	HBA:HBD Molar Ratio (X_3_)	Water (%) (X_4_)	Extraction Method (X_5_)	Extraction Time (min) (X_6_)	Y_1_ (TPC)	Y_2_ (TFC)	Y_3_ (TAA)
		Independent Variables with Coded Values						Dependent Variables (Mean ^#^ ± SD, n = 3)		
N1	6	L-Pro	PGL	2	30	UTE *	10	169.53 ± 0.16	7.49 ± 0.02	81.07 ± 0.06
N2	24	L-Pro	PGL	1	50	UTE	10	221.87 ± 0.03	7.64 ± 0.06	198.05 ± 0.11
N3	10	L-Pro	PGL	1	40	UTE	5	217.87 ± 0.05	7.79 ± 0.13	107.88 ± 0.03
N4	36	L-Pro	PGL	1.5	30	UTE	5	238.70 ± 0.09	9.07 ± 0.04	161.98 ± 0.08
N5	38	ChChl	PGL	2	50	UTE	5	181.37 ± 0.20	4.49 ± 0.02	90.34 ± 0.03
N6	25	ChChl	PGL	1	30	UTE	10	153.03 ± 0.05	4.24 ± 0.05	128.54 ± 0.55
N7	8	L-Pro	LA	2	50	UTE	5	74.37 ± 0.04	2.95 ± 0.00	16.57 ± 0.04
N8	11	L-Pro	LA	2	50	UTE	10	218.37 ± 0.06	6.92 ± 0.01	8.86 ± 2.01
N9	39	L-Pro	LA	1	30	UTE	7.5	256.53 ± 0.09	6.97 ± 0.01	95.17 ± 0.04
N10	16	ChChl	LA	2	30	UTE	5	165.70 ± 0.02	4.61 ± 0.03	155.59 ± 0.06
N11	20	ChChl	LA	1	50	UTE	5	353.20 ± 0.09	4.82 ± 0.01	206.41 ± 0.05
N12	9	ChChl	LA	2	30	UTE	10	195.53 ± 0.10	5.06 ± 0.01	178.05 ± 0.05
N13	2	ChChl	LA	1	50	UTE	10	213.70 ± 0.01	5.27 ± 0.01	117.39 ± 0.06
N14	27	L-Pro	Glu	2	30	UTE	5	227.70 ± 0.04	6.89 ± 0.04	115.01 ± 0.04
N15	15	L-Pro	Glu	1	50	UTE	5	191.87 ± 0.02	7.40 ± 0.05	198.21 ± 0.01
N16	32	L-Pro	Glu	1	30	UTE	10	255.20 ± 0.04	7.73 ± 0.01	108.71 ± 0.01
N17	29	ChChl	Glu	1	30	UTE	5	226.03 ± 0.03	6.53 ± 0.01	238.05 ± 0.04
N18	3	ChChl	Glu	2	50	UTE	10	166.03 ± 0.12	5.63 ± 0.04	212.48 ± 0.02
N19	28	ChChl	Glu	1.5	40	UTE	7.5	194.53 ± 0.03	5.41 ± 0.03	218.54 ± 0.15
N20	26	L-Pro	PGL	2	50	UAE **	5	205.20 ± 0.01	8.38 ± 0.01	239.03 ± 0.04
N21	33	L-Pro	PGL	1	30	UAE	10	223.87 ± 0.02	8.66 ± 0.01	252.97 ± 0.03
N22	30	ChChl	PGL	2	30	UAE	5	153.03 ± 0.05	4.93 ± 0.02	174.77 ± 0.03
N23	7	ChChl	PGL	1	50	UAE	5	173.20 ± 0.03	5.64 ± 0.02	181.49 ± 0.03
N24	5	ChChl	PGL	2	50	UAE	10	371.37 ± 0.03	13.83 ± 0.02	255.59 ± 0.03
N25	35	L-Pro	LA	2	30	UAE	5	456.03 ± 0.02	14.29 ± 0.05	227.56 ± 0.05
N26	14	L-Pro	LA	1	50	UAE	5	408.03 ± 0.02	12.85 ± 0.04	243.46 ± 0.02
N27	22	L-Pro	LA	2	30	UAE	10	414.70 ± 2.91	10.95 ± 0.02	203.13 ± 0.01
N28	21	L-Pro	LA	1	50	UAE	10	365.53 ± 0.04	10.95 ± 0.03	235.92 ± 0.03
N29	18	ChChl	LA	1	30	UAE	5	370.20 ± 0.06	9.74 ± 0.02	184.77 ± 0.02
N30	19	ChChl	LA	1	30	UAE	10	318.53 ± 0.01	8.48 ± 0.03	205.43 ± 0.02
N31	31	ChChl	LA	2	50	UAE	7.5	341.37 ± 0.07	8.96 ± 0.01	229.03 ± 0.06
N32	12	L-Pro	Glu	1	30	UAE	5	424.70 ± 0.37	14.55 ± 0.04	256.08 ± 0.03
N33	17	L-Pro	Glu	2	50	UAE	10	319.20 ± 0.03	11.61 ± 0.09	251.82 ± 0.03
N34	34	ChChl	Glu	2	50	UAE	5	353.37 ± 0.04	10.55 ± 0.06	249.53 ± 0.03
N35	4	ChChl	Glu	2	30	UAE	10	408.20 ± 0.14	11.84 ± 0.08	259.36 ± 0.04
N36	13	ChChl	Glu	1	50	UAE	10	443.03 ± 0.11	11.15 ± 0.09	263.13 ± 0.01
N37	23	ChChl	Glu	1.5	40	UAE	7.5	490.53 ± 0.10	11.78 ± 0.06	257.23 ± 0.07
N38	37	ChChl	Glu	1.5	40	UAE	7.5	390.70 ± 0.10	9.53 ± 0.02	261.16 ± 0.01
N39	1	ChChl	Glu	1.5	40	UAE	10	447.87 ± 0.02	12.45 ± 0.03	255.75 ± 0.07

* UTE—ultra-turrax extraction; ** UAE—ultrasound-assisted extraction; ChChl—choline chloride; Glu—*β*-D-glucose; LA—lactic acid; L-Pro—L-proline; PGL—propylene glycol; Y_1_—total phenolic content (TPC), expressed as milligrams of gallic acid equivalents per gram of dry weight: mg GAE/g dw; Y_2_—total flavonoid content (TFC), expressed as milligrams of quercetin equivalents per gram of dry weight: mg QE/g dw; Y_3_—total antioxidant activity measured by the DPPH method (TAA-DPPH), expressed as milligrams of Trolox equivalents per gram of dry weight: mg TE/g dw. ^#^ Values are presented as the mean ± standard deviation (SD) of three replicates (n = 3).

**Table 2:** The fitting parameters for the assessed responses.

Quantifiable Responses	R^2^	Q^2^	Model Validity	Reproducibility
Total Phenolic Content (Y_1_)	0.855	0.735	0.895	0.787
Total Flavonoid Content (Y_2_)	0.870	0.808	0.948	0.738
Total Antioxidant Activity (Y_3_)	0.872	0.720	0.418	0.996

**Table 3:** ANOVA test results regarding the significance of the TPC, TFC, and TAA models.

Quantifiable Responses	*p* Value	
	Regression	Lack of Fit
Total Phenolic Content (Y_1_)	<0.001	0.660
Total Flavonoid Content (Y_2_)	<0.001	0.813
Total Antioxidant Activity (Y_3_)	<0.001	0.098

**Table 4:** Centralized results of negative and positive effects identified as statistically significant (*p* < 0.05) for the factors studied.

Independent Factor	Number and Type of Effects	
X_1_	Negative	Positive
Choline chloride	7	4
L-proline	4	7
X_2_	Negative	Positive
Glucose	6	11
Lactic acid	11	7
Propylene glycol	9	10
X_3_	Negative	Positive
Combination ratio	0	3
X_4_	Negative	Positive
Water ratio	0	6
X_5_	Negative	Positive
Ultra-turrax extraction	13	10
Ultrasound-assisted extraction	10	13
X_6_	Negative	Positive
Extraction time	1	4

**Table 5:** Results for the optimal extracts obtained from *Ribes nigrum* leaves under the provided optimal extraction conditions.

**Sample Name**	**HBA—** **L-pro** **(X_1_)**	**HBD—LA** **(X_2_)**	**HBA:HBD** **Molar Ratio (X_3_)**	**Water (%)** **(X_4_)**	**Extraction Method (X_5_)**	**Extraction** **Time (min) (X_6_)**	**Dependent Variables**			
								**Determined**	**Predicted**	**Recovery**
							TPC (Y_1_)	379.37 ± 0.06	371.76	102.05%
							TFC (Y_2_)	12.39 ± 0.02	11.85	104.52%
Optimal extract	1124	1	1124	49.977	UAE *	5	TAA (Y_3_)	216.43 ± 0.03	229.79	94.18%

* UAE—ultrasound-assisted extraction; L-Pro—L-proline; LA—lactic acid; Y_1_—total phenolic content (TPC), expressed as milligrams of gallic acid equivalents per gram of dry weight: mg GAE/g dw; Y_2_—total flavonoid content (TFC), expressed as milligrams of quercetin equivalents per gram of dry weight: mg QE/g dw; Y_3_—total antioxidant activity measured by the DPPH method (TAA-DPPH), expressed as milligrams of Trolox equivalents per gram of dry weight: mg TE/g dw.

**Table 6:** Phenolic acid compounds analyzed in blackcurrant leaf extracts.

Exp. Name	Run Order	Hydroxycinnamic Acids (μg/g dw)					Hydroxybenzoic Acids (μg/g dw)		
		*p*-Coumaric Acid	Caffeic Acid	Chlorogenic Acid	4-*O*-Caffeoylquinic Acid	Gallic Acid	Protocatechuic Acid	Gentisic Acid	Vanillic Acid
N1	6	5.82	4.58	28.81	6.60	12.39	13.57	<LOQ	0.54
N2	24	9.13	13.08	54.10	15.40	8.35	7.09	<LOQ	0.94
N3	10	5.51	8.50	52.21	13.49	7.18	6.50	<LOQ	0.79
N4	36	5.82	5.45	52.97	12.72	7.19	6.79	<LOQ	0.68
N5	38	5.21	4.14	52.59	13.11	4.76	4.90	<LOQ	0.67
N6	25	3.11	2.61	45.42	10.05	3.64	4.09	<LOQ	0.62
N7	8	<LOQ	2.40	46.93	11.19	0.14	0.07	<LOQ	0.01
N8	11	2.20	3.92	57.87	14.25	6.70	1.66	<LOQ	0.52
N9	39	3.11	3.49	55.99	16.17	5.68	1.69	<LOQ	0.25
N10	16	<LOQ	1.96	50.70	11.96	2.97	1.13	<LOQ	0.20
N11	20	<LOQ	3.05	57.12	11.96	4.83	1.17	<LOQ	0.14
N12	9	<LOQ	2.40	51.84	12.34	3.37	1.14	<LOQ	0.19
N13	2	<LOQ	3.05	58.25	11.58	4.96	1.41	<LOQ	0.11
N14	27	4.31	7.41	43.15	11.58	6.07	4.96	3.04	0.19
N15	15	7.02	11.34	52.97	14.64	8.36	6.00	<LOQ	0.31
N16	32	1.60	4.14	21.64	5.84	3.31	3.26	<LOQ	0.13
N17	29	7.92	5.67	59.01	15.02	7.06	6.16	<LOQ	0.35
N18	3	6.12	6.54	54.85	15.02	6.00	5.70	<LOQ	0.37
N19	28	9.43	9.59	66.93	16.93	7.04	6.92	<LOQ	0.43
N20	26	12.14	13.96	56.36	21.14	9.47	7.89	<LOQ	0.43
N21	33	ND	2.18	50.33	ND	7.78	7.14	5.84	0.36
N22	30	ND	1.52	45.04	ND	3.63	4.01	<LOQ	0.35
N23	7	ND	2.61	55.99	ND	5.08	5.19	<LOQ	0.42
N24	5	<LOQ	<LOQ	3992.21	ND	3.02	19.77	4.44	0.07
N25	35	<LOQ	<LOQ	2799.42	6.60	3.28	4.52	<LOQ	0.05
N26	14	<LOQ	<LOQ	2968.52	8.51	3.52	5.62	<LOQ	0.03
N27	22	<LOQ	<LOQ	1742.13	6.60	10.61	13.29	<LOQ	0.05
N28	21	<LOQ	<LOQ	2386.47	9.28	10.08	12.61	<LOQ	0.02
N29	18	<LOQ	<LOQ	2675.61	7.75	3.49	7.65	<LOQ	ND
N30	19	<LOQ	<LOQ	2364.20	8.90	5.09	9.41	<LOQ	ND
N31	31	<LOQ	<LOQ	2179.24	6.98	5.59	13.96	11.45	ND
N32	12	<LOQ	70.67	1757.23	ND	4.71	20.71	<LOQ	ND
N33	17	<LOQ	129.78	2022.97	ND	7.18	28.21	5.14	ND
N34	34	<LOQ	<LOQ	2142.62	ND	4.32	19.45	3.74	ND
N35	4	<LOQ	<LOQ	3404.49	ND	2.25	14.99	<LOQ	ND
N36	13	<LOQ	<LOQ	2524.24	ND	1.91	13.40	<LOQ	ND
N37	23	<LOQ	<LOQ	3798.95	ND	2.55	18.51	<LOQ	ND
N38	37	<LOQ	<LOQ	3044.01	ND	3.90	20.32	<LOQ	ND
N39	1	<LOQ	<LOQ	3942.01	ND	3.17	19.97	<LOQ	ND

<LOQ—below the quantification limit of the analytical method; ND—not determined.

**Table 7:** Bioactive compounds from the flavanol class analyzed in blackcurrant leaf extracts.

Exp. Name	Run Order	Catechins (µg/g dw)				Procyanidins (µg/g dw)						
		Epicatechin	Catechin	EGC	EGCG	B3	B1	B4	B2	C2	C1	A1
N1	6	2.78	4.68	24.15	0.42	5.44	1.84	1.89	0.98	ND	ND	4.38
N2	24	2.87	3.14	35.76	0.57	1.52	1.14	1.43	2.29	ND	1.65	5.17
N3	10	2.79	3.07	36.94	0.57	1.44	0.92	1.06	0.06	ND	1.91	2.20
N4	36	2.63	2.87	37.77	0.59	1.61	0.84	0.60	0.09	ND	2.09	ND
N5	38	2.44	2.66	38.43	0.43	1.06	0.45	0.01	0.42	ND	1.90	ND
N6	25	2.36	2.78	36.31	0.63	0.54	0.15	0.03	1.13	ND	1.78	ND
N7	8	0.07	0.07	1.41	ND	0.82	0.13	0.05	0.66	ND	1.17	ND
N8	11	2.58	2.07	38.27	ND	1.10	0.14	0.03	0.63	ND	0.73	ND
N9	39	1.88	1.33	34.42	ND	1.13	0.16	0.01	0.43	ND	0.70	ND
N10	16	1.50	0.92	26.06	ND	0.45	0.06	0.02	0.69	ND	1.31	ND
N11	20	1.59	0.80	26.40	0.31	0.25	0.03	0.06	0.59	ND	0.81	ND
N12	9	1.19	0.77	24.80	0.36	0.33	0.05	0.03	0.57	ND	0.95	ND
N13	2	1.21	0.86	27.72	0.24	1.09	0.19	0.04	0.50	ND	0.83	ND
N14	27	1.49	1.10	27.96	0.38	0.26	0.02	0.01	1.25	ND	1.42	ND
N15	15	1.60	1.29	29.57	0.62	0.52	0.08	0.04	1.67	ND	2.14	ND
N16	32	0.64	1.32	18.07	0.31	0.09	0.01	0.01	0.72	ND	0.97	ND
N17	29	1.81	1.95	38.50	0.55	0.68	0.16	0.08	1.72	ND	1.97	ND
N18	3	1.62	1.93	35.08	0.44	0.24	0.05	0.03	1.93	ND	2.02	ND
N19	28	2.02	2.25	43.03	0.46	1.28	0.40	0.60	2.19	ND	1.90	ND
N20	26	1.66	2.26	39.93	0.47	0.19	0.06	0.27	1.98	ND	1.74	ND
N21	33	1.86	2.19	38.33	0.57	0.13	0.04	0.40	1.76	ND	1.72	ND
N22	30	1.50	2.24	33.00	0.39	0.11	0.06	0.51	1.68	ND	1.59	ND
N23	7	1.63	2.21	34.54	0.50	0.21	0.17	0.89	2.60	ND	2.55	ND
N24	5	36.58	2.32	35.06	ND	2.67	16.30	15.02	150.05	19.97	217.76	3.07
N25	35	20.05	9.85	15.41	ND	36.84	20.25	19.52	83.79	14.18	119.35	29.23
N26	14	19.04	11.92	11.31	ND	40.08	21.72	18.07	61.98	9.53	81.33	34.30
N27	22	6.83	4.77	19.44	ND	23.12	13.53	10.89	30.30	5.42	58.87	18.05
N28	21	8.82	6.72	21.55	ND	31.24	19.51	15.20	40.19	7.55	58.25	20.02
N29	18	9.55	5.89	16.71	ND	30.76	20.46	16.72	63.50	6.07	73.72	15.04
N30	19	10.75	4.82	20.13	ND	26.37	16.63	12.24	53.23	5.69	69.37	16.03
N31	31	13.04	6.10	18.62	ND	26.51	13.86	12.81	42.84	4.67	75.98	18.08
N32	12	11.37	5.89	4.99	ND	36.46	19.33	12.09	46.58	6.68	58.58	20.09
N33	17	13.06	5.97	2.85	ND	36.84	15.63	12.03	49.14	7.41	79.44	19.70
N34	34	17.30	8.16	18.70	ND	42.48	24.31	15.48	64.90	6.76	99.17	20.56
N35	4	25.82	3.60	35.11	ND	15.92	18.52	13.25	89.16	10.65	142.78	10.63
N36	13	17.39	1.70	22.47	ND	5.83	13.41	8.07	72.63	7.56	123.67	ND
N37	23	23.03	1.69	30.16	ND	8.63	17.86	11.22	91.33	9.15	158.25	ND
N38	37	16.39	6.81	26.10	ND	28.68	25.17	16.75	77.74	8.90	120.99	13.11
N39	1	26.45	5.90	35.07	ND	22.28	21.60	13.88	87.39	8.68	150.54	13.03

EGC—epigallocatechin; EGCG—epigallocatechin gallate; ND—not determined.

**Table 8:** Flavonol class bioactive compounds analyzed by LC-MS in blackcurrant leaf extracts (data are expressed as μg/g dw).

Exp. Name	Run Order	Hyperoside	Isoquercetin	Rutin	Quercitrin	Quercetol	Kaempferol-3-O-rhamnoside	Kaempferol
N1	6	34.67	98.07	62.18	60.72	<LOQ	<LOQ	1.42
N2	24	9.27	443.27	106.71	188.78	9.13	<LOQ	4.73
N3	10	9.27	330.77	100.03	146.72	<LOQ	<LOQ	3.74
N4	36	9.27	199.01	94.10	89.70	<LOQ	<LOQ	2.08
N5	38	8.24	151.24	98.55	55.11	<LOQ	<LOQ	2.08
N6	25	8.76	91.90	90.39	32.67	<LOQ	<LOQ	1.75
N7	8	8.76	88.82	89.64	32.67	<LOQ	<LOQ	3.07
N8	11	10.83	111.94	108.20	42.96	<LOQ	<LOQ	5.40
N9	39	10.83	108.86	105.97	39.22	<LOQ	<LOQ	3.74
N10	16	8.76	87.28	97.07	33.61	<LOQ	<LOQ	<LOQ
N11	20	11.35	97.30	105.97	35.48	<LOQ	<LOQ	3.41
N12	9	9.79	90.36	97.81	32.67	<LOQ	<LOQ	<LOQ
N13	2	10.83	101.92	110.43	37.35	<LOQ	<LOQ	2.08
N14	27	8.76	361.59	77.03	155.13	4.45	<LOQ	2.08
N15	15	10.83	466.39	108.94	195.33	5.00	<LOQ	3.07
N16	32	4.09	148.92	42.14	61.65	<LOQ	<LOQ	0.75
N17	29	9.27	216.73	111.17	79.41	<LOQ	<LOQ	2.41
N18	3	7.72	306.11	104.49	113.06	<LOQ	<LOQ	2.41
N19	28	11.87	303.03	126.01	110.26	<LOQ	<LOQ	2.41
N20	26	7.20	537.28	123.79	230.85	12.43	<LOQ	7.05
N21	33	9.27	148.15	111.17	ND	ND	ND	1.42
N22	30	6.16	81.89	86.67	ND	ND	ND	<LOQ
N23	7	6.16	180.52	88.90	ND	ND	ND	2.08
N24	5	216.59	94.99	<LOQ	89.70	ND	ND	1.08
N25	35	189.64	77.26	<LOQ	69.13	ND	ND	<LOQ
N26	14	213.48	84.20	<LOQ	85.96	ND	ND	<LOQ
N27	22	117.08	77.26	<LOQ	97.17	ND	ND	1.42
N28	21	163.73	94.99	<LOQ	123.35	ND	ND	2.41
N29	18	169.95	81.89	<LOQ	87.83	ND	ND	<LOQ
N30	19	155.43	84.97	<LOQ	105.59	ND	ND	1.08
N31	31	144.03	76.49	<LOQ	95.30	ND	ND	<LOQ
N32	12	126.93	60.31	<LOQ	60.72	ND	ND	<LOQ
N33	17	148.18	84.97	<LOQ	48.56	ND	ND	1.08
N34	34	133.67	61.85	<LOQ	63.52	ND	ND	<LOQ
N35	4	173.06	74.95	<LOQ	70.06	ND	ND	<LOQ
N36	13	129.00	54.92	ND	28.00	ND	ND	<LOQ
N37	23	198.45	73.41	<LOQ	41.09	ND	ND	<LOQ
N38	37	165.80	74.18	<LOQ	70.06	ND	ND	<LOQ
N39	1	203.12	71.87	<LOQ	63.52	ND	ND	<LOQ

<LOQ—below the quantification limit of the analytical method; ND—not determined.

**Table 9:** Number average of intermolecular hydrogen bonds of chlorogenic acid in the systems using L-proline as the HBA.

System	L-Proline	HBD		Water	Total
1	0.85	propylene glycol	0.81	4.64	6.29
2	0.88		0.32	5.93	7.13
3	1.11		0.71	5.67	7.50
4	1.67		0.91	5.88	8.46
20	1.67		0.75	5.28	7.70
21	0.78		0.59	5.71	7.09
14	1.32	*β*-D-glucose	1.48	4.23	7.04
15	0.77		1.15	6.93	8.85
16, 32	0.70		1.58	5.03	7.31
33	0.85		0.92	6.18	7.96
7, 8	2.56	R-lactic acid:L-lactic acid 1:1	0.38	5.63	8.37
9	5.25		0.71	5.36	10.91
25, 27	1.20		0.36	4.76	6.18
26, 28	0.71		0.50	6.01	6.95

**Table 10:** Number of average intermolecular hydrogen bonds of chlorogenic acid in the systems using choline chloride as the HBA.

System	Choline Chloride	HBD		Water	Total
5, 24	0.19	propylene glycol	0.48	0.48	5.69
6	0.16		1.75	1.75	2.80
22	0.24		1.12	1.12	2.94
23	0.11		0.79	0.79	5.52
17	0.18	β-D-glucose	1.06	5.20	6.44
18	0.23		1.34	4.37	5.93
19, 37, 38, 39	0.13		1.53	5.07	6.73
34	0.23		2.02	3.71	5.96
35	0.12		1.57	5.97	7.65
36	0.17		1.41	6.29	7.87
10, 12	0.37	R-lactic acid:L-lactic acid 1:1	0.74	3.44	4.00
11, 13	0.16		0.51	5.68	6.08
29	0.26		0.55	7.07	7.62
31	0.22		1.10	3.87	4.68

**Table 11:** The average value of interaction energy between chlorogenic acid and the mixture used as a solvent in the systems using L-proline as the HBA (kJ/mol).

System	HBD	Electrostatic	van der Waals	Total
1	propylene glycol	−223.19	−136.44	−359.63
2		−252.66	−125.21	−377.87
3		−244.67	−123.71	−368.38
4		−245.29	−125.11	−370.39
20		−235.75	−137.19	−372.94
21		−231.43	−134.24	−365.66
14	*β*-D-glucose	−248.23	−143.59	−391.81
15		−283.17	−116.00	−399.17
16, 32		−245.78	−142.86	−388.64
33		−271.26	−126.12	−397.38
7, 8	R-lactic acid:L-lactic acid 1:1	−244.47	−123.67	−368.14
9		−256.32	−128.88	−385.19
25, 27		−273.96	−125.81	−399.77
26, 28		−245.53	−133.32	−378.84

**Table 12:** The average value of interaction energy between chlorogenic acid and the mixture used as a solvent in the systems using choline chloride as the HBA (kJ/mol).

System	HBD	Electrostatic	van der Waals	Total
5, 24	propylene glycol	−254.81	−104.46	−359.27
6		−245.68	−114.23	−359.91
22		−242.93	−119.58	−362.51
23		−254.73	−108.24	−362.97
17	*β*-D-glucose	−260.27	−124.32	−384.59
18		−262.15	−119.50	−381.65
19, 37, 38, 39		−262.98	−123.18	−386.16
34		−282.65	−109.53	−392.18
35		−280.62	−133.55	−414.17
36		−273.78	−125.60	−399.39
10, 12	R-lactic acid:L-lactic acid 1:1	−252.92	−116.58	−369.50
11, 13		−255.20	−109.46	−364.66
29		−259.69	−114.95	−374.64
31		−299.86	−95.13	−394.99

**Table 13:** The results of the disk diffusion test assay for the investigated Gram-positive, Gram-negative, and fungi strains of NaDES mixtures alone and blackcurrant leaf extracts obtained in NaDESs, and the extract obtained under optimal conditions (OE), versus standard drugs. The results are expressed as the diameter of the inhibition area (mm).

Sample Code	Gram-Positive Bacterial Strains				Gram-Negative Bacterial Strains			Fungi
Blank (NaDES Mixture)	*Staphylococcus aureus*	*Enterococcus* *faecalis*	*Streptococcus pyogenes*	*Listeria monocytogenes*	*Escherichia coli*	*Klebsiella pneumoniae*	*Pseudomonas aeruginosa*	*Candida albicans*
24	0	0	0	0	0	0	0	0
25	10.18	8	23.64	15.04	10.85	10.8	10.6	6.69
26	10.42	6.85	14.22	10.12	9.26	9.72	10.28	0
27	16	6.75	18.59	10.25	12.2	14.8	16.61	8.12
28	10.67	8.59	20.79	11.93	11	11.24	11.88	0
29	10.49	10	22	13.44	13.07	11.28	13.88	8.67
30	13	10.07	23.68	14.46	13	14.04	19.03	10.07
31	0	0	0	0	0	0	0	0
32	0	0	0	0	0	0	0	0
33	0	0	0	0	0	0	0	0
34	0	0	0	0	0	0	0	0
35	0	0	0	0	0	0	0	0
36	0	0	0	0	0	0	0	0
37	0	0	0	0	0	0	0	0
38	0	0	0	0	0	0	0	0
39	0	0	0	0	0	0	0	7.67
A	24.64	15.34	22.67	12.67	-	-	-	-
G	-	-	-	-	18.81	19.07	24.63	-
K	-	-	-	-	-	-	-	32.44
Blank of OE	10.54	16.83	16.86	17.25	15.36	14.59	16.57	0
**NaDES Extract**								
24	6.59	7.23	10.04	7.19	0	9.63	0	13.69
25	14.02	9.19	14.04	9.61	9.3	10.95	16.03	8.86
26	13.18	9.04	16.21	17.84	11.34	9.95	13.14	0
27	6.82	9.14	13.51	7.08	0	0	8.18	0
28	10.98	10.19	15.51	16.96	9.96	10.8	10.15	0
29	13.69	12.91	18.93	21.87	11.71	11.47	18.12	9.87
30	14.13	13.13	19.04	18.84	12.74	13.1	16.81	9.73
31	7.65	7.6	10.05	9.09	9.04	7.89	0	0
32	0	0	8.77	7.02	0	0	0	24.78
33	7.71	6.68	11.32	7.32	0	0	0	20.88
34	7.07	7.6	10.06	7.08	0	0	0	13.06
35	6.38	8.02	10.87	6.69	0	0	11.48	28.04
36	7.11	0	10.18	7.08	0	0	11.95	21.52
37	6.75	6.89	10.2	7.67	0	0	10.68	22.98
38	7.18	7.57	9.32	6.38	0	0	9.94	25.42
39	0	7.27	12.31	7.78	0	0	0	0
A	24.57	15.29	21.48	11.35	-	-	-	-
G	-	-	-	-	19.34	18.24	24.89	-
K	-	-	-	-	-	-	-	32.26
OE	11.67	15.55	15.69	17.13	16.98	16.73	15.16	6.22

Legend: A—Amoxicillin; G—Gentamicin; K—Ketoconazole; OE—extract obtained under optimal conditions.

**Table 14:** The results from the in vitro quantitative evaluation of NaDES mixtures alone, blackcurrant leaf extracts obtained in NaDESs, and the extract obtained under optimal conditions (OE).

Sample Code	Gram-Positive Bacterial Strains												Gram-Negative Bacterial Strains									Fungi		
Blank (NaDES mixture)	*Staphylococcus* *aureus*			*Enterococcus* *faecalis*			*Streptococcus* *pyogenes*			*Listeria* *monocytogenes*			*Escherichia* *coli*			*Klebsiella* *pneumoniae*			*Pseudomonas* *aeruginosa*			*Candida* *albicans*		
	MIC	MBC	MIC index	MIC	MBC	MIC index	MIC	MBC	MIC index	MIC	MBC	MIC index	MIC	MBC	MIC index	MIC	MBC	MIC index	MIC	MBC	MIC index	MIC	MBC	MIC index
24	1/4	1/2	2	1/32	1/4	8	1/64	1/32	2	1/32	1/16	2	NA	NA	NA	1/4	1/1	4	NA	NA	NA	NA	NA	NA
25	1/128	1/32	4	1/128	1/32	4	1/128	1/64	4	1/128	1/64	2	1/64	1/64	1	1/64	1/64	1	1/128	1/128	1	1/8	1/8	1
26	1/64	1/32	2	1/64	1/32	2	1/64	1/32	2	1/256	1/32	8	1/64	1/32	2	1/64	1/64	1	1/64	1/64	1	NA	NA	NA
27	1/128	1/64	2	1/128	1/64	2	1/128	1/64	2	1/128	1/64	2	1/64	1/64	1	1/64	1/64	1	1/128	1/128	1	1/8	1/4	2
28	1/128	1/64	2	1/128	1/64	2	1/128	1/64	2	1/128	1/64	2	1/64	1/64	1	1/64	1/64	1	1/128	1/64	2	NA	NA	NA
29	1/128	1/64	2	1/128	1/64	2	1/128	1/64	2	1/128	1/64	2	1/128	1/64	2	1/64	1/16	4	1/128	1/128	1	1/8	1/8	1
30	1/128	1/64	2	1/128	1/64	2	1/128	1/64	2	1/128	1/64	2	1/128	1/64	2	1/64	1/16	4	1/128	1/128	1	1/8	1/8	1
31	1/8	1/2	4	1/8	1/4	2	1/32	1/16	2	1/32	1/32	1	NA	NA	NA	NA	NA	NA	NA	NA	NA	NA	NA	NA
32	NA	NA	NA	NA	NA	NA	NA	NA	NA	NA	NA	NA	NA	NA	NA	NA	NA	NA	NA	NA	NA	NA	NA	NA
33	NA	NA	NA	NA	NA	NA	NA	NA	NA	NA	NA	NA	NA	NA	NA	NA	NA	NA	NA	NA	NA	NA	NA	NA
34	NA	NA	NA	NA	NA	NA	NA	NA	NA	NA	NA	NA	NA	NA	NA	NA	NA	NA	NA	NA	NA	NA	NA	NA
35	NA	NA	NA	NA	NA	NA	NA	NA	NA	NA	NA	NA	NA	NA	NA	NA	NA	NA	NA	NA	NA	NA	NA	NA
36	NA	NA	NA	NA	NA	NA	NA	NA	NA	NA	NA	NA	NA	NA	NA	NA	NA	NA	NA	NA	NA	NA	NA	NA
37	NA	NA	NA	NA	NA	NA	NA	NA	NA	NA	NA	NA	NA	NA	NA	NA	NA	NA	NA	NA	NA	NA	NA	NA
38	NA	NA	NA	NA	NA	NA	NA	NA	NA	NA	NA	NA	NA	NA	NA	NA	NA	NA	NA	NA	NA	NA	NA	NA
39	NA	NA	NA	NA	NA	NA	NA	NA	NA	NA	NA	NA	NA	NA	NA	NA	NA	NA	NA	NA	NA	1/2	1/2	1
Blank of OE	1/32	1/16	2	1/64	1/32	2	1/256	1/32	8	1/256	1/32	8	1/64	1/32	2	1/64	1/32	2	1/64	1/64	1	1/8	1/4	2
**NaDES** **extract**	**MIC**	**MBC**	**MIC index**	**MIC**	**MBC**	**MIC index**	**MIC**	**MBC**	**MIC index**	**MIC**	**MBC**	**MIC index**	**MIC**	**MBC**	**MIC index**	**MIC**	**MBC**	**MIC index**	**MIC**	**MBC**	**MIC index**	**MIC**	**MBC**	**MIC index**
24	1/64	1/16	4	1/512	1/32	16	1/256	1/32	8	1/256	1/32	8	1/128	1/16	8	1/16	1/4	4	NA	NA	NA	1/8	1/2	4
25	1/128	1/32	4	1/256	1/64	4	1/512	1/64	8	1/512	1/64	8	1/128	1/16	8	1/64	1/32	2	1/256	1/256	1	1/4	1/4	1
26	1/128	1/64	2	1/128	1/64	2	1/512	1/64	8	1/512	1/64	8	1/64	1/64	1	1/64	1/64	1	1/256	1/256	1	NA	NA	NA
27	1/256	1/16	8	1/256	1/64	4	1/256	1/32	8	1/256	1/32	8	1/128	1/32	4	1/64	1/64	1	1/64	1/32	2	NA	NA	NA
28	1/256	1/64	4	1/128	1/64	2	1/512	1/64	8	1/256	1/64	4	1/64	1/64	1	1/64	1/64	1	1/128	1/64	2	NA	NA	NA
29	1/128	1/64	2	1/128	1/64	2	1/256	1/64	4	1/512	1/64	8	1/128	1/64	2	1/128	1/64	2	1/128	1/64	2	1/4	1/4	1
30	1/128	1/64	2	1/256	1/64	4	1/512	1/64	8	1/256	1/128	2	1/128	1/64	2	1/128	1/64	2	1/128	1/64	2	1/8	1/8	1
31	1/64	1/16	4	1/256	1/32	8	1/512	1/64	8	1/256	1/32	8	1/32	1/32	1	1/32	1/32	1	NA	NA	NA	NA	NA	NA
32	1/128	1/16	8	1/256	1/32	8	1/128	1/64	2	1/256	1/32	8	NA	NA	NA	NA	NA	NA	NA	NA	NA	1/2	1/1	2
33	1/64	1/8	8	1/512	1/64	8	1/512	1/64	8	1/256	1/32	8	NA	NA	NA	NA	NA	NA	NA	NA	NA	1/4	1/2	2
34	1/64	1/16	4	1/512	1/64	8	1/256	1/32	8	1/256	1/32	8	NA	NA	NA	NA	NA	NA	NA	NA	NA	1/8	1/2	4
35	1/64	1/8	8	1/512	1/64	8	1/256	1/32	8	1/512	1/64	8	1/64	1/8	4	1/16	1/4	4	1/128	1/64	2	1/128	1/32	4
36	1/128	1/16	8	1/512	1/64	8	1/512	1/64	8	1/512	1/64	8	1/64	1/8	4	NA	NA	NA	1/128	1/64	2	1/256	1/32	8
37	1/128	1/16	8	1/512	1/64	8	1/512	1/64	8	1/512	1/64	8	NA	NA	NA	NA	NA	NA	1/128	1/64	2	1/256	1/32	8
38	1/128	1/16	8	1/512	1/64	8	1/512	1/64	8	1/512	1/32	16	NA	NA	NA	NA	NA	NA	1/128	1/64	2	1/512	1/64	8
39	1/128	1/16	8	1/512	1/64	8	1/2048	1/128	16	1/256	1/32	8	NA	NA	NA	NA	NA	NA	NA	NA	NA	NA	NA	NA
OE	1/64	1/32	2	1/128	1/64	2	1/256	1/64	4	1/256	1/64	4	1/64	1/64	1	1/64	1/32	2	1/128	1/64	2	1/8	1/4	2

Legend: MIC—minimum inhibitory concentration; MBC—minimum bactericidal concentration; MIC index—calculated as MIC/MBC is used to assess the effectiveness of antimicrobial combinations compared to the effectiveness of the individual agents used alone. NA—not active. OE—extract obtained under optimal conditions.

**Table 15:** Independent and dependent variables of the experimental design.

Variables	Level		
	−1	0	1
Independent variables (factors)			
HBA (X_1_)	L-Pro		ChChl
HBD (X_2_)	PGL	LA	Glu
HBA:HBD molar ratio (X_3_)			
Water (%) (X_4_)			
Extraction method (X_5_)	UTE	-	UAE
Extraction time (X_6_)	5	7.5	10
Dependent variables (responses)			
Total phenolic content (TPC, mg GAE/g dw ^1^) (Y_1_) Total flavonoid content (TFC, mg QE/g dw ^2^) (Y_2_) Total antioxidant activity (DPPH, mg TE/g dw ^3^) (Y_3_)			

^1^—mg gallic acid equivalents per g of dry weight (mg GAE/g dw); ^2^—mg quercetin equivalents per g of dry weight (mg QE/g dw); ^3^—mg Trolox equivalents per g dry weight (mg TE/g dw); ChChl -choline chloride; L-Pro: l-proline; PGL: propylene glycol; LA: lactic acid; Glu: glucose.

**Table 16:** The number of molecules in each system using L-proline as the HBA.

System	L-proline	HBD		Water
1	1554	propylene glycol	777	5669
2	634		634	6733
3	881		881	6238
4	1425		950	5626
20	799		400	6801
21	1221		1221	5558
14	1096	*β*-D-glucose	548	5356
15	380		380	6239
16, 32	775		775	5450
33	543		271	6186
7, 8	866	R-lactic acid:L-lactic acid 1:1	433	7702
9	1307		1307	6386
25, 27	1694		847	6459
26, 28	672		672	7657

**Table 17:** The number of molecules in each system using choline chloride as the HBA.

System	Choline	Chloride	HBD		Water
5, 24	748	748	propylene glycol	374	7379
6	1191	1191		1191	6118
22	1483	1483		742	6275
23	608	608		608	7284
17	832	832	*β*-D-glucose	832	6336
18	561	561		280	7159
19, 37, 38, 39	709	709		473	6819
34	561	561		280	7159
35	1148	1148		574	6278
36	405	405		405	7191
10, 12	1357	1357	R-lactic acid:L-lactic acid 1:1	678	5965
11, 13	542	542		542	6916
29	1071	1071		1071	5858
31	680	680		340	6979

## Data Availability

Data are contained within the article.

## Supplementary Materials

The following supporting information can be downloaded at https://www.mdpi.com/article/10.3390/antibiotics13121118/s1, Table S1. Bioactive compounds for which negative and positive effects were identified as statistically significant (*p* < 0.05) for the evaluated factors; Figure S1. Score (a) and loading (b) plots of the OPLS-DA model comparing Group 1 (systems 25, 26, 28, 29, and 30) and Group 2 (remaining extracts); Table S2. The setpoint and predicted properties of the optimal extract; Table S3: Identification and quantification of polyphenolic compounds in *Ribes nigrum* L. leaves.
