# Supplementary material for: Antimicrobial Effectiveness of Ribes nigrum L. Leaf Extracts Prepared in Natural Deep Eutectic Solvents (NaDESs)
Source: Antibiotics (Basel). 2024 Nov 22;13(12):1118. doi: 10.3390/antibiotics13121118 (PMC11672518; doi:10.3390/antibiotics13121118)
Supplement: Supplementary file 1 [file antibiotics-13-01118-s001.zip › antibiotics-3247407-supplementary.pdf]

antibiotics

Article

Antimicrobial Effectiveness of *Ribes nigrum* L. Leaf Extracts Prepared in Natural Deep Eutectic Solvents (NaDESs)

Maria-Beatrice Solcan [1], Ana-Maria Vlase [2,*], Gabriel Marc [3], Dana Muntean [4], Tibor Casian [4], George Cosmin Nadăș [5], Cristiana Ștefania Novac [5], Daniela-Saveta Popa [1] and Laurian Vlase [4]

Supplementary material

2. Results

2.1.2. The Influence of Experimental Conditions on Dependent Variables

In Table S1 are presented the individual bioactive compounds for which the extraction yield was statistically significantly influenced, either positively or negatively, by the independent factors evaluated.

Table S1. Bioactive compounds for which negative and positive effects were identified as statistically significant ($p<0.05$) for the evaluated factors

**Independent Factor**	**Number and Type of Effects for Bioactive Compounds in NaDES extracts from *Ribes nigrum* leaves**			
X_1	Negative		Positive	
Choline chloride	7	caffeic acid, *p*-coumaric acid, isoquercitrin, quercetol, kaempferol, gallic acid, vanillic acid	4	chlorogenic acid, EGC, EGCG, procyanidin B2
L-proline	4	chlorogenic acid, EGC, EGCG, procyanidin B2	7	caffeic acid, *p*-coumaric acid, isoquercitrin, quercetol, kaempferol, gallic acid, vanillic acid
X_2	Negative		Positive	
Glucose	6	4-*O*-caffeoilquinic acid, rutin, kaempferol, vanillic acid, EGC, EGCG	11	chlorogenic acid, *p*-coumaric acid, hyperoside, isoquercitrin, quercitrin, quercetol, protocatechuic acid, procyanidins B1, B2, C1, and C2
Lactic acid	11	caffeic acid, *p*-coumaric acid, isoquercitrin, rutin, quercetol, kaempferol, gallic acid, protocatechuic acid, vanillic acid, ECG, EGCG	7	chlorogenic acid, 4-*O*-caffeoilquinic acid, hyperoside, quercitrin, procyanidins B1, B3, and C2
Propylene glycol	9	chlorogenic acid, 4-*O*-caffeoilquinic acid, hyperoside, quercitrin, procyanidins B1, B2, B3, C1 and C2	10	caffeic acid, *p*-coumaric acid, isoquercitrin, rutin, kaempferol, gallic acid, protocatechuic acid, vanillic acid, EGC, EGCG
X_3	Negative		Positive	
Combination ratio	0	-	3	gentisic acid, hyperoside, quercitrin

Independent Factor	Number and Type of Effects for Bioactive Compounds in NaDES extracts from *Ribes nigrum* leaves			
X_4	Negative		Positive	
Water ratio	0	-	6	gentisic acid, isoquercitrin, quercitrin, quercetol, kaempferol, procyanidin B2
X_5	Negative		Positive	
Ultra-Turrax extraction	13	gentisic acid, chlorogenic acid, hyperoside, epicatechin, catechin, protocatechuic acid, procyanidins A1, B1, B2, B3, B4, C1, and C2	10	caffeic acid, 4-*O*-caffeoilquinic acid, *p*-coumaric acid, isoquercitrin, rutin, quercitrin, kaempferol, vanillic acid, EGC, EGCG
Ultrasound-assisted extraction	10	caffeic acid, 4-*O*-caffeoilquinic acid, *p*-coumaric acid, isoquercitrin, rutin, quercitrin, kaempferol, vanillic acid, EGC, EGCG	13	gentisic acid, chlorogenic acid, hyperoside, epicatechin, catechin, protocatechuic acid, procyanidins A1, B1, B2, B3, B4, C1, and C2
X_6	Negative		Positive	
Extraction time	1	isoquercitrin	4	hyperoside, kaempferol, protocatechuic acid, procyanidin B2

Legend: EGC - Epigallocatechin; EGCG – Epigallocatechin gallate.

2.5.2. Antimicrobial Activity - In Vitro Quantitative Study

The optimization procedure of the extraction process was guided by the results of the antimicrobial activity. The extracts were categorized into two groups based on their antimicrobial activity, and OPLS-DA models were employed to identify key phytochemical compounds with discriminatory power. Variables were scaled to unit variance prior to fitting the models.

The OPLS-DA model comparing Group 1 (extracts 25, 26, 28, 29, 30) and Group 2 (the remaining extracts) demonstrated strong predictive capacity ($Q^2 = 0.57$), with 17.2% of the composition variation explained by inter-group differences. A score scatter plot clearly distinguished Group 1 from other extracts (Figure S1a). Group 1's superior antimicrobial properties were attributed to its higher content of compounds like chlorogenic acid, hyperoside, catechin, B3, B1, B4, C2, A1, as well as total phenolic content (TPC) and total flavonoid content (TFC) (Figure S1b). To this respect, the extraction process was optimized by maximizing all these constituents.

a

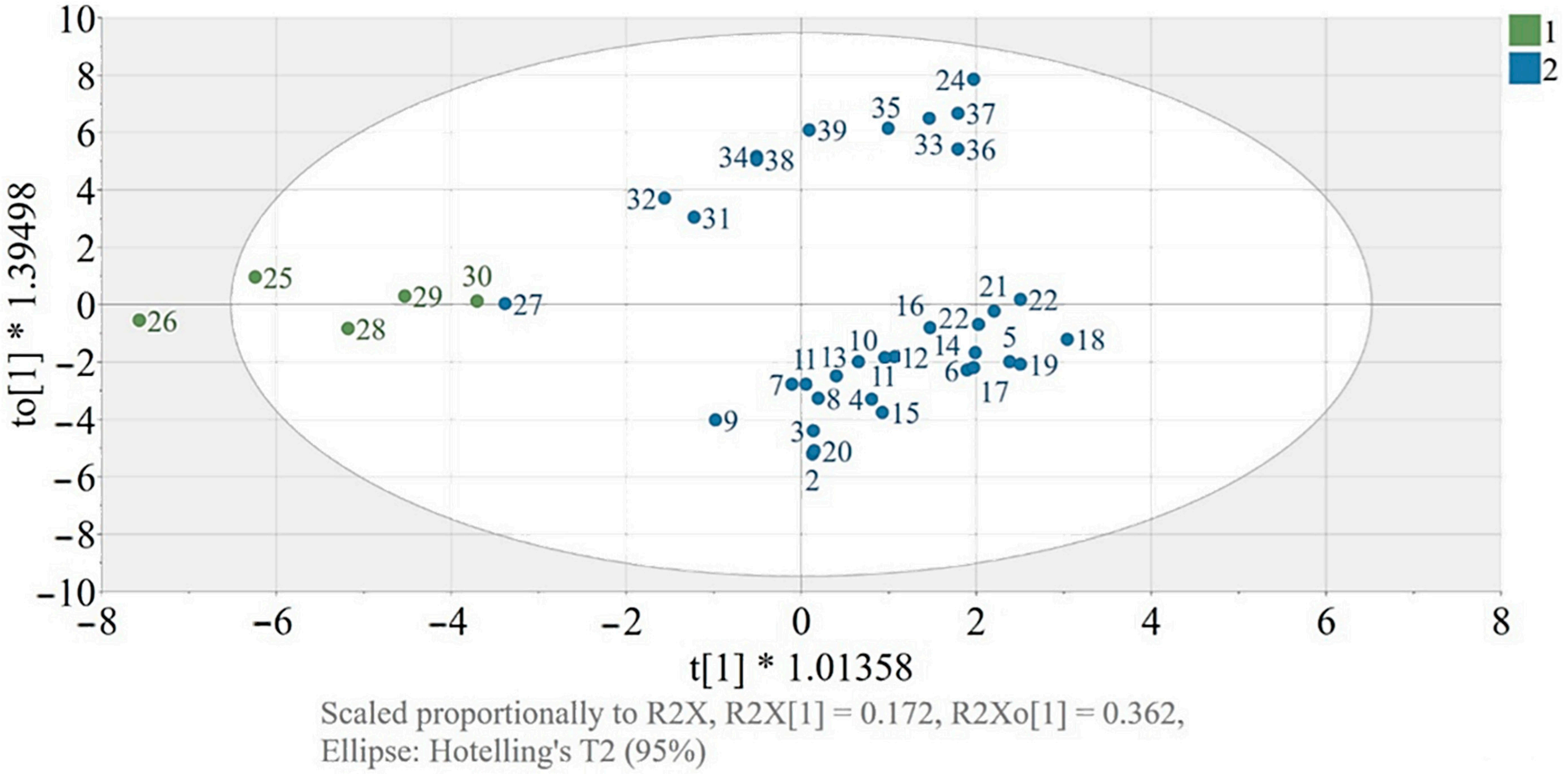
to[1] * 1.39498
t[1] * 1.01358
1
2
Scaled proportionally to R2X, R2X[1] = 0.172, R2Xo[1] = 0.362,
Ellipse: Hotelling's T2 (95%)

b

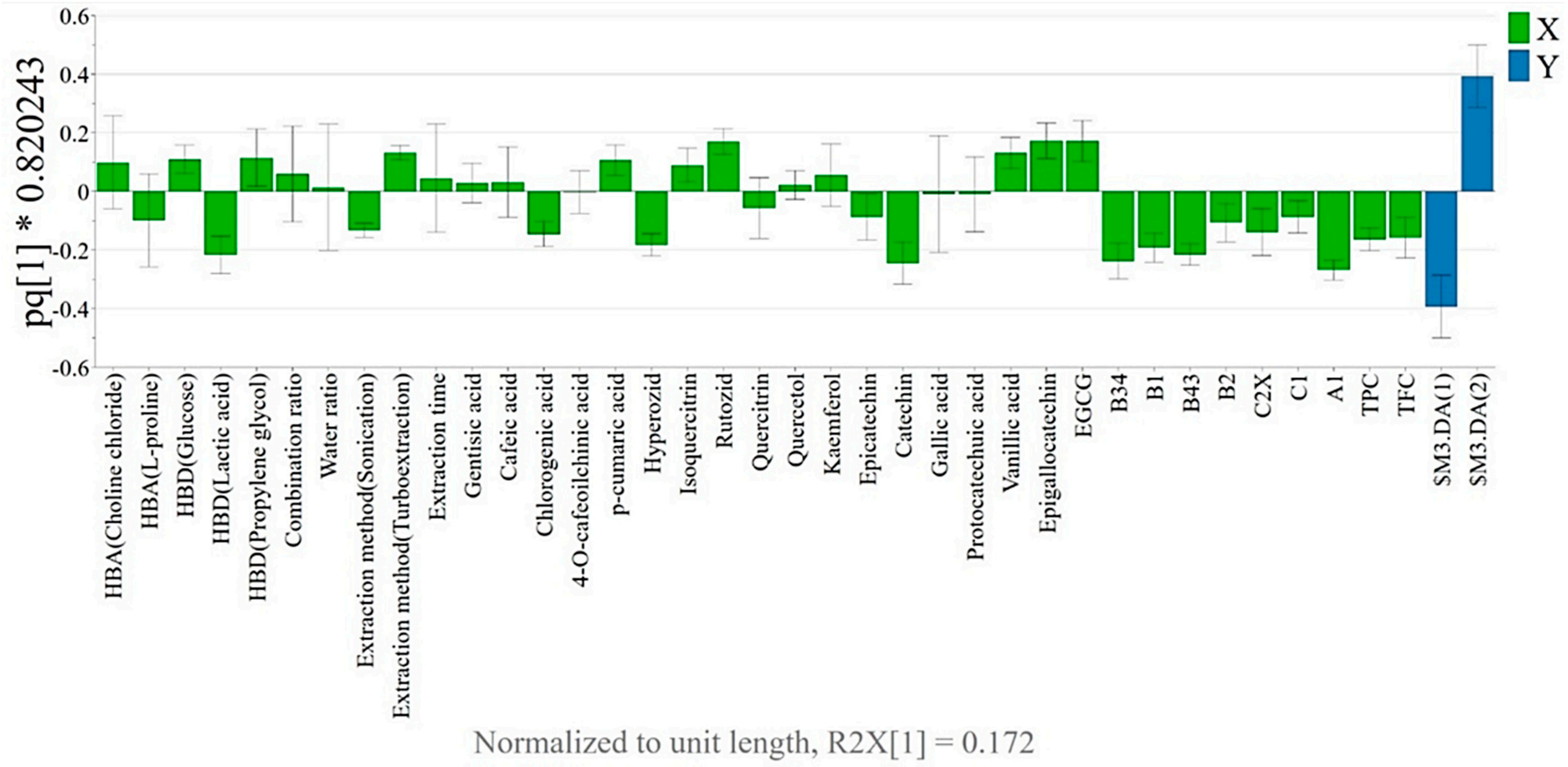

Figure S1. Score (a) and loading (b) plots of OPLS-DA model comparing Group 1 (25, 26, 28, 29, 30) and Group 2 (remaining extracts)

The setpoint predicted by the optimization software for the previously defined objectives is presented in Table S2.

Table S2. The setpoint and predicted properties of the optimal extract

Factor	Value	Response	Predicted value	Observed value	Recovery (%)
HBA	L-proline	TPC (mg GAE/g dw)	371.76	379.37	102.05
HBD	Lactic acid	TFC (mg QE/g dw)	11.85	12.39	104.52
Molar combination ratio (HBA:HBD)	1.1247	Chlorogenic acid (µg/g dw)	2555.73	2832.75	110.84
Water ratio (%)	49.9771	Hyperoside (µg/g dw)	220.46	224.83	101.98
Extraction method	UAE	Catechin (µg/g dw)	6.77	6.98	103.12
Extraction time (min)	5.00334	Procyanidins (µg/g dw)			
		B3	31.87	31.78	99.72
		B1	17.72	17.09	96.46
		B4	24.09	23.19	96.26
		C2	6.75	7.11	105.31
		A1	22.32	21.67	97.11

Legend: HBA – hydrogen bond acceptor; HBD – hydrogen bond donor; UAE – ultrasound-assisted extraction; TPC – total phenolic content, expressed as expressed as milligrams of gallic acid equivalents per gram of dry weight – mg GAE/g dw; TFC – total flavonoid content, expressed as milligrams of quercetin equivalents per gram of dry weight – mg QE/g dw.

The similarity of setpoint settings between the two optimization objectives confirms that the optimal preparation conditions for extracting the phytochemical constituents with the best extraction yield are matched with the initial ones.

3. Discussion

3.1. Quantitative determination of total bioactive compounds and antioxidant activity

Additional information regarding the phytochemical composition of extracts prepared using classical solvents for the same plant matrix can be found in Supplementary Table S3.

antibiotics

MDPI

Table S3. Identification and quantification of polyphenolic compounds in *Ribes nigrum* L. leaves.

Ref.	Origin	Extract	Analytical Method	Compounds	Amount
Stevic et al., 2010 [37]	Čačanska crna cultivar (June, 1000 m), Serbia	Methanolic extract + acid hydrolysis of the flavonol glycosides	HPLC-DAD	TPC	40,1 ± 2,1 mg GAE/g dw
				Flavonols	
				Quercetin	84 ± 2,4 mg/g dw
				Kaempferol	43,6 ± 1,6 mg/g dw
				Myricetin	9,5 ± 0,4 mg/g dw
Oszmiański et al., 2011 [5]	Titania cultivar, Poland	Water extract	LC-ESI/MS<br>HPLC-DAD	*Hydroxycinnamic derivatives*	
				Chlorogenic acid (3-*O*-caffeoylquinic acid)	1.15%
				Neochlorogenic acid (5-*O*-caffeoylquinic acid)	0.1%
				Cryptochlorogenic acid (4-*O*-caffeoylquinic acid)	0.11%
				Quercetin derivatives	11.21%
				Rutin (Quercetin-3-*O*-rutinoside)	0.33%
				Hyperoside (Quercetin-3-*O*-galactoside)	2.52%
				Quercetin-3-*O*-glucoronide	0.92%
				Quercetin-3-(6″-malonyl)-glucoside	1.91%
				Quercetin-3-*O*-glucosyl-6″-acetate	5.53%
				Kaempferol derivatives	
				Kaempferol-3-*O*-glucoside	0.46%
				Kaempferol-3-*O*-galactoside	0.41%
Tabart et al., 2011 [2]	Noir de Bourgogne cultivar (August), Belgium	Acetone extracts		TPC	46,0 ± 8,4 mg CAE/g fw
				TFC	2,05 ± 0,34 mg QE/g fw
				TA	381 ± 72 µg KuE/g fw
				Total Flavanols	1,47 ± 0,87 µg CE/g fw
			HPLC-DAD	*Phenolic acids*	

antibiotics

MDPI

Ref.	Origin	Extract	Analytical Method	Compounds	Amount
				Gallic acid	1015 ± 54 µg/g fw
				Gentisic acid	about 1900 µg/g fw (*)
				Anthocyanidins	
				Delphinidin	85 ± 14 µg/g fw
				Petunidin	514 ± 152 µg/g fw
				Cyanidin	65 ± 19 µg/g fw
				Peonidin	35 ± 7 µg/g fw
				Total anthocyanins	381 ± 72 µg KuE/g fw
				Flavan-3-ols	
				Epigallocatechin	150 ± 86 µg/g fw
				Gallocatechin	382 ± 132 µg/g fw
				Catechin	19 ± 9 µg/g fw
				Epicatechin	2,5 ± 1,6 µg/g fw
				Total flavan-3-ols	1.47 ± 0.87 mg CE/g fw
				Flavonols	
				Myricetin	139 ± 47 µg/g fw
				Quercetin	778 ± 203 µg/g fw
				Kaempferol	322 ± 151 µg/g fw
				Total flavonols	2.05 ± 0.34 µg QE/g fw
		Specific extraction for phenolic acids in methanol and hydrolysis		*Phenolic acids*	
				Gallic acid	1883 ± 90 µg/g fw
				Gentisic acid	traces (*)
				p-Hydroxibenzoic acid	1572 ± 32 µg/g fw
				p-Coumaric acid	about 200 µg/g fw (*)
				Anthocyanidins	
				Delphinidin	88 ± 18 µg/g fw

antibiotics MDPI

Ref.	Origin	Extract	Analytical Method	Compounds	Amount
		Specific extraction for anthocyanins in methanol		Petunidin	1181 ± 115 µg/g fw
				Cyanidin	363 ± 138 µg/g fw
				Peonidin	133 ± 75 µg/g fw
				Pelargonidin	258 ± 196 µg/g fw
				Malvidin	178 ± 119 µg/g fw
				Total anthocyanins	429 ± 87 µg KuE/g fw
				Flavan-3-ols	
				Total flavan-3-ols	9.0 ± 4.4 µg CE/g fw
		Specific extraction for flavan-3-ols in acetone			
Chrzanowski et al., 2012 [46]	Bona cultivar (July), Poland	Methanolic extraction	HPLC-DAD	*Hydroxycinnamic acids and derivatives*	
				p-Coumaric acid	13.6 ± 0.4 µg/g dw
				o-Coumaric acid	187.5 ± 4.5 µg/g dw
				Caffeic acid	40.6 ± 1.3 µg/g dw
				Chlorogenic acid	20.9 ± 1.2 µg/g dw
				Hydroxybenzoic acids and derivatives	
				p-Hydroxybenzoic acid	467.6 ± 34.8 µg/g dw
				Benzoic acid	84.6 ± 2.3 µg/g dw
				Vanilic acid	68.7 ± 2.5 µg/g dw
				Syringic acid	128.5 ± 2.7 µg/g dw
				Sum of phenolic acids	1011.9 ± 49.6 µg/g dw
Vagiri et al., 2012 [15]	Cultivated blackcurrant (September), Sweden	Ethanolic extracts + UAE		TPC	89–97 mg GAE/g dw
			HPLC-DAD<br>HPLC-ESI-MS	*Flavonols*	
				Myricetin malonyl-glucoside	
				Myricetin malonyl-glucoside (isomer)	
				Quercetin-3-*O*-rutinoside	
				Quercetin-3-*O*-galactoside	

Ref.	Origin	Extract	Analytical Method	Compounds	Amount
				Quercetin-3-*O*-glucoside<br>Quercetin-3-6-malonyl-glucoside<br>Kaempferol-3-*O*-rutinoside<br>Kaempferol-3-*O*-glucoside<br>Isorhamnetin-3-*O*-rutinoside<br>Isorhamnetin-3-*O*-glucoside<br>Kaempferol-malonylglucoside<br>Kaempferol-malonylglucoside (isomer)<br><br>*Flavan-3-ols*<br>Epigallocatechin<br>Catechin<br>Epicatechin<br><br>*Phenolic acid derivatives*<br>Neochlorogenic acid<br>Chlorogenic acid<br><br>*Anthocyanins*<br>Delphinidin-3-*O*-glucoside<br>Delphinidin-3-*O*-rutinoside<br>Cyanidin-3-*O*-glucoside<br>Cyanidin-3-*O*-rutinoside	NQ
Teleszko and Wojdylo, 2015 [42]	Titania[a], Tiben[b], and Tisel[c] cultivares, Poland	Methanolic extracts + UAE	UPLC-PDA and FLD<br>UPLC-ESI-MS	Polymeric proanthocyanidins<br><br>Mono-, di-, and oligomeric flavan-3-ols<br>Phenolic acids<br><br>Flavonols	429.95 mg/100 g dw (792.09[a]; 238.95[b]; 258.87[c])<br>194.79 mg/100 g dw (200.87[a]; 179.01[b]; 204.65[c])<br>52.94 mg/100 g dw (33.69[a]; 60.08[b]; 65.06[c])<br>700.68 mg/100 g dw

Ref.	Origin	Extract	Analytical Method	Compounds	Amount
				Total polyphenols	(673.31[a]; 636.44[b]; 792.28[c])<br>1,378.36 mg/100 g dw<br>(1699.96[a]; 1114.48[b]; 1320.86[c])
Nowak et al., 2016 [39]	Cultivated blackcurrant (September), Poland	Water extraction	LC-ESI-MS<br>HPLC-DAD	TPC<br><br>*Phenolic acids and derivatives*<br>Gallic acid<br>Neochlorogenic acid<br>Caffeic acid derivative<br>Syryngin glucoside<br>Chlorogenic acid<br><br>*Flavonoids*<br>Epigallocatechin<br>Quercetin glicoside<br>Kaempferol galactoside<br>Kaempferol rutinoside<br>Kaempferol glucoside (astragaline)	2,17 mg GAE/g<br><br><br>0.53 ± 0.02<br>0.14 ± 0.01<br>traces<br>traces<br>traces<br><br><br>traces<br>0.61 ± 0.03<br>traces<br>0.23 ± 0.03<br>traces
Raudsepp et al., 2019 [50]	Pamyati Vavilova cultivar, Estonia	Infusions in 20% and 96% ethanol	HPLC-UV<br>UHPLC-MS	The most abundant compounds:<br>Catechin gallate<br>Chlorogenic acid I<br>Dihydro ferulic acid rhamnoside<br>Chlorogenic acid II<br>Ferulic acid derivative<br>Coumaryl quinic acid<br>Coumaroylquinic acid pentoside<br>Myricetin-glucoside<br>Quercetin-3-rutinoside syn. Rutin<br>Quercetin glucoside<br>Quercetin acetylglucoside<br>Kaempferol rutinoside	NQ

Ref.	Origin	Extract	Analytical Method	Compounds	Amount
				Kaempferol-3-*O*-glucoside<br>Kaempferol acetylglucoside<br>Isorhamnetin acetylglucoside<br>Chrysophanol glucoside<br>Oxylipin<br>Oxylipin 9S,12S,13S-trihydroxy-10E-octadecenoic acid (9,12,13-TriHOME)	
D'Urso et al., 2020 [47]	Italy	Infusion<br>Hydroethanolic extract (HEE)[a]<br>HEE + UAE[b]<br>Methanolic extract (ME)[c]<br>ME + UAE	LC-ESI/LTQ-Orbitrap/MS<br>UPLC-ESI-QTrap-MS/MS	31 phenolic compounds of which major compounds:<br>Quinic acid<br>Catechin<br>Chlorogenic acid<br>Quercetin glucoside<br>Rutin<br>Quercetin<br>Myricetin-galactoside<br>Coumaroyl quinic acid<br>Quercetin diglucuronide<br>Quercetin acetyl glucoside<br>Myricetin<br>Kaempferol diglucoside<br>Kaempferol hexoside<br>Kaempferol hexoside2<br>Kaempferol acetyl glucoside<br>Kaempferol rutinoside	206.9 ± 2.5 mg/100 g dw[b]<br>2.87 ± 1.3 mg/100 g dw[a]<br>3.5 ± 0.3 mg/100 g dw[a]<br>26.2 ± 0.8 mg/100 g dw[a]<br>10.8 ± 1.4 mg/100 g dw[c]<br>4.8 ± 0.1 mg/100 g dw[a]<br>6.3 ± 0.4 mg/100 g dw[b]<br>152.5 ± 1.5 mg/100 g dw[b]<br>0.33 ± 0.04 mg/100 g dw[a]<br>0.26 ± 0.03 mg/100 g dw[a,b]<br>8.9 ± 3.8 mg/100 g dw[c]<br>0.61 ± 0.2 mg/100 g dw[a,b]<br>1.55 ± 0.31 mg/100 g dw[b]<br>5.76 ± 0.57 mg/100 g dw[a]<br>2.9 ± 0.30 mg/100 g dw[a]<br>1.9 ± 0.25 mg/100 g dw[b]
Magnavacca et al, 2021 [40]	Wild flora (July, 1600-1650 m), Armenia	Water extract	LC-ESI-LTQ-MS/MS	TPC<br>*Flavonoids*<br>Kaempferol aglycone<br>Kaempferol-7-glucoside	17.260 ± 0.473 mg GAE/g extract (13.8%)<br>wt.% in extract:<br>2.9%<br>3.8%

antibiotics MDPI

Ref.	Origin	Extract	Analytical Method	Compounds	Amount
				Kaempferol-3-glucoside	1.3%
				Quercetin aglycone	1.9%
				Hyperoside	0.8%
				Rutin	1.6%
				Chlorogenic acid	1.5%
Hovhannisyan et al., 2022 [41]	Wild flora (July, 1600-1650 m), Armenia	Ethanolic extracts	LC-Q-Orbitrap HR-MS	TPC	84.1 ± 1.6 mg GAE/g dw
				TFC	35.42 ± 1.52 mg QE/g dw
				Hydroxycinnamic acids derivatives:	
				Caffeoyl quinic acid isomers	
				Coumaric acid derivatives	
				Flavonols:	
				Quercetin rutinoside	
				Quercetin hexoside	
				Quercetin glucuronide	
				Quercetin malonylglucoside	
				Quercitrin	
				Flavan-3-ols:	
				(+)-Catechin	NQ
				(-)-Epicatechin	
				(+)-Gallocatechin or (-)-Epigallocatechin	
				Galloylated and digalloylated dimeric B-type proanthocyanidins	
				Trimeric and tetrameric A-type pro-anthocyanidins	
				Other compounds:	
				Lariciresinol glucoside (lignan)	
				Musizin (nepodin) derivatives (naphthol group)	
				Marmesin glucoside (furocoumarin)	
				Marmesin acetyl glucoside (psoralen)	

Ref.	Origin	Extract	Analytical Method	Compounds	Amount
Paunovic et al., 2022 [38]	Cultivar Titania (July, 242 m), Serbia	Ethanolic extracts + UAE	HPLC-UV	TPC<br>TFC<br>Condensed tannins<br>Gallotannins<br>TAC<br><br>*Individual phenolic compounds:*<br>Ellagic acid<br>Ferulic acid<br>Caffeic acid<br>*p*-Coumaric acid<br>Quercetin<br>Myricetin<br>Kaempferol	2.17 mg GAE/g dw<br>1.36 mg RuE/g dw<br>1.71 mg GAE/g dw<br>1.25 mg GAE/g dw<br>1.96 mg ascorbic acid/g dw<br><br><br>0.120 ± 0.01 mg/100 g dw<br>0.970 ± 0.07 mg/100 g dw<br>0.523 ± 0.04 mg/100 g dw<br>0.270 ± 0.02 mg/100 g dw<br>0.712 ± 0.05 mg/100 g dw<br>0.241 ± 0.02 mg/100 g dw<br>0.116 ± 0.01 mg/100 g dw
Babayan and Sahakyan, 2023 [36]	Wild flora (July, 1600-1650 m), Armenia	Ethanolic extracts		TPC<br>TFC	167.15 ± 7.29 mg GAE/g dw<br>49.99 ± 0.86 mg QE/g dw

Legend: CAE – chlorogenic acid equivalents; CE – catechin equivalents; DAD – diode array detector; dw – dry weight; ESI – electrospray source; FLD – fluorescence detector; fw – frozen weight; GAE – gallic acid equivalents; HPLC – high performance liquid chromatography; HR – high resolution; KuE - kuromanin (cyanidin-3-glucosid) equivalents; LC – liquid chromatography; LQT - linear trap quadrupole; MS – mass spectrometry; NQ – non quantified; Q – quadrupole; QE – quercetin equivalents; PDA – photodiode array detector; RuE – rutin equivalents; TA – total anthocyanins; TAC – total antioxidant capacity; TFC – total flavonoid content; TPC – total phenolic content; UAE – ultrasound assisted extraction; (*) – quantities taken from figures (not exactly stated).
